# Strongly Coupled 2D Transition Metal Chalcogenide-MXene-Carbonaceous Nanoribbon Heterostructures with Ultrafast Ion Transport for Boosting Sodium/Potassium Ions Storage

**DOI:** 10.1007/s40820-021-00623-5

**Published:** 2021-04-22

**Authors:** Junming Cao, Junzhi Li, Dongdong Li, Zeyu Yuan, Yuming Zhang, Valerii Shulga, Ziqi Sun, Wei Han

**Affiliations:** 1 Sino-Russian International Joint Laboratory for Clean Energy and Energy Conversion Technology, International Center of Future Science, College of Physics, Jilin University, Changchun, 130012 People’s Republic of China; 2 Centre for Materials Science, School of Chemistry and Physics, Queensland University of Technology (QUT), 2 George Street, Brisbane, QLD 4001 Australia; 3 Key Laboratory of Advanced Energy Materials Chemistry (Ministry of Education), College of Chemistry, Nankai University, Tianjin, 300071 People’s Republic of China

**Keywords:** Ti_3_C_2_T_*x*_ MXene, Heterostructure, Transition metal chalcogenide, Sodium and potassium-ions batteries, DFT calculation

## Abstract

Combining with the advantages of two-dimensional (2D) nanomaterials, MXenes have shown great potential in next generation rechargeable batteries. Similar with other 2D materials, MXenes generally suffer severe self-agglomeration, low capacity, and unsatisfied durability, particularly for larger sodium/potassium ions, compromising their practical values. In this work, a novel ternary heterostructure self-assembled from transition metal selenides (MSe, M = Cu, Ni, and Co), MXene nanosheets and N-rich carbonaceous nanoribbons (CNRibs) with ultrafast ion transport properties is designed for sluggish sodium-ion (SIB) and potassium-ion (PIB) batteries. Benefiting from the diverse chemical characteristics, the positively charged MSe anchored onto the electronegative hydroxy (–OH) functionalized MXene surfaces through electrostatic adsorption, while the fungal-derived CNRibs bonded with the other side of MXene through amino bridging and hydrogen bonds. This unique MXene-based heterostructure prevents the restacking of 2D materials, increases the intrinsic conductivity, and most importantly, provides ultrafast interfacial ion transport pathways and extra surficial and interfacial storage sites, and thus, boosts the high-rate storage performances in SIB and PIB applications. Both the quantitatively kinetic analysis and the density functional theory (DFT) calculations revealed that the interfacial ion transport is several orders higher than that of the pristine MXenes, which delivered much enhanced Na^+^ (536.3 mAh g^−1^@ 0.1 A g^−1^) and K^+^ (305.6 mAh g^−1^@ 1.0 A g^−1^ ) storage capabilities and excellent long-term cycling stability. Therefore, this work provides new insights into 2D materials engineering and low-cost, but kinetically sluggish post-Li batteries.

**Supplementary Information:**

The online version contains supplementary material available at 10.1007/s40820-021-00623-5.

## Highlights

- Unique “Janus” interfacial assemble strategy of 2D MXene nanosheets was proposed firstly.
- Ternary heterostructure consisting of high capacity transitional metal chalcogenide, high conductive 2D MXene and N rich fungal carbonaceous matrix was achieved for larger radius Na/K ions storages.
- The highly accessible surfaces and interfaces of the strongly coupled 2D based ternary heterostructures provide superb surficial pseudocapacitive storages for both Na and K ions with low energy barriers was verified.

**Supplementary Information:**

The online version contains supplementary material available at 10.1007/s40820-021-00623-5.

## Graphical abstract

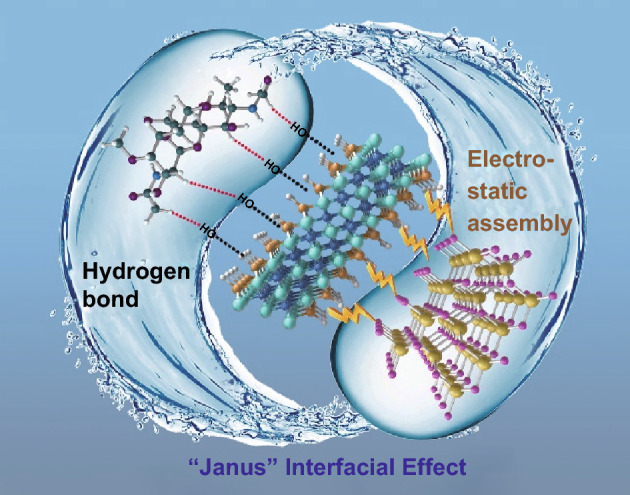

## Introduction

Exploring low-cost next generation sodium-ion (SIB) and potassium-ion (PIB) batteries are of great prospects to alleviate the increasing dependence on limited Li resources in the earth’s crust aroused by the snowballing use of lithium-ion battery (LIBs) [1, 2]. The current performances of SIBs and PIBs, however, are yet unsatisfying, due to the sluggish kinetics resulting from the larger radius of Na^+^ and K^+^ during the electrochemical alloying and dealloying reactions [3, 4]. In order to facilitate the development toward commercialization, it is a priority to investigate high-performance electrode materials for both SIBs and PIBs, which should be more favorable for larger alkali ions storage and transport [5].

Two dimensional (2D) nanomaterials greatly promote the progress of energy storage technologies and the application of rechargeable batteries, thanks to their higher interfacial chemical activity, shortened diffusion pathways of ions, and enhanced in-plane carrier or charge mobilization kinetics, in comparison to the materials in other dimensions [6]. MXenes, an emerging 2D transitional metal carbides or nitrides, have drawn intensive research attentions in energy storages by virtue of their unique physicochemical properties, such as rich surficial groups, metallic conductivity, and chemical compatibility [7–10]. Nevertheless, the deficiencies of easy self-aggregation, unsatisfying ions accessibility, and low theoretical capacity are generally inevitable for MXenes, because of the hydrogen bonds or van der Walls interactions between the laminated adjacent layers. Particularly, the performance is yet far to be satisfied when used as the anode of the batteries with larger alkali-metal ions [3, 11]. It is thus requested the design of advanced heterostructures to address the above-mentioned challenges in the electrochemical energy storage applications [12, 13].

In this work, we proposed a novel 2D strongly coupled ternary heterostructure self-assembled from transition metal selenides (MSe, M = Cu, Ni, and Co), MXene nanoflakes (MXene), and fungal-derived N-rich carbonaceous nanoribbons (CNRibs) to address the issues of restacking, relatively low capacity, and rapid capacity decay of MXenes-based anode in SIBs and PIBs [14, 15]. By taking the full advantages of rich chemistry on the surfaces of MXene, the electropositive transition metal ions (Cu^2+^, Ni^2+^, and Co^2+^) were anchored onto one side of the negatively charged MXene nanoflakes to form transition metal selenide, and the CNRib was attached onto the other side of MXene through amino bridging groups and hydrogen bonds. Compared to other heterostructures with unique interfacial coupling states, this ternary heterostructure could deliver a “Janus” interfacial effect with different bonding behaviors [16]. The interfaces were also further tailored by depositing MSe with different cations, including Cu, Ni, and Co, to search the most suitable coupling for desired electrostatic adsorption and improved ions transport and storage capability. In this heterostructure, CNRibs feature high electric conductivity, which can act as high-speed ways for charges transfer, and MXene serves as the core active counterpart for high-efficient ion storages, as demonstrated by previous studies, and works as the backbone together with CNRibs to maintain the structural stabilization, while TMC further endows high lattice ion storage capacity and enhanced surficial pseudocapacitive ion storage. It is worth noting that these constituent components are jointed through strong chemical bonds instead of usually the weak van de Waals connected 2D heterostructure. Our previous work has demonstrated that strongly coupled 2D-2D TiO_2_-g-C_3_N_4_ heterostructures with significant interfacial electronic coupling and transfer behaviors can offer much enhanced photo-induced organic synthesis and degradation reactions, which sheds light on designing strongly couples 2D heterostructures to further expand the advantages of this promising class of materials [17]. We thus expected that, through architecting the strongly coupled TMC-MXene-CNRib heterostructures, the ternary interfaces composed of property-complementary constituents will provide not only structural integrity and accessibility but also ultrafast ion transport pathways and abundant active ion storage sites to achieve high rate ions diffusion and high capacity storage for large sodium and potassium ions. We also hope that this work can inspire the design of novel electrode structures with ultrafast interfacial ion transport properties to boost the performance of the post-Li batteries based on emerging MXenes and other 2D nanomaterials.

## Experimental

### Chemicals

400 mesh MAX phase (Ti_3_AlC_2_) powder was purchased from 11 Technology Co., Ltd., China, 99% ascorbic acid (C_6_H_8_O_6_), 49 wt% hydrofluoric acid (HF), 99% copper (II) acetate monohydrate (C_4_H_6_CuO_4_·H_2_O, AR), 99.9% cobalt acetate tetrahydrate (C_4_H_6_CoO_4_·4H_2_O, AR) and selenium powder (Se) were obtained from Aladdin Reagent, China. 99 + % Nickel acetate hydrate (C_4_H_6_NiO_4_·4H_2_O, AR) was obtained from Alfa Aesar, China. 40 wt% tetramethylammonium hydroxide (TMAOH) was obtained from Beijing inno-chem, China. Deionized water was self-supply in our laboratory.

### Preparation of Ti_3_C_2_T_***x***_ MXenes

2.0 g Ti_3_AlC_2_ MAX powder was slowly added into 20 mL 40% HF to prevent oxidation process resulting from the overheat exothermic reaction. Following by 6 h etching progress under the constant temperature of 35 ℃ stirring ceaselessly, the multilayered Ti_3_C_2_T_*x*_ MXene was obtained. After mixing with 1.0 g C_6_H_8_O_6_ in 30 mL TMAOH, the mixture was undergone a hydrothermal treatment for 24 h under 140 ℃. When initial two centrifugation and rinsing are finished, the following supernatants was collected for subsequent use, the concentration of dispersion is about 7 mg mL^−1^.

### Culture, Calcination and Selenylation Process

Firstly, *Aspergillus niger* was grown after a culture process with a nutrient solution consist of 10 g glucose and 8 g peptone, under 35 ℃ for 3 days in an orbital shaker incubator. Above obtained supernatant (about 300 ml) was mixed with fungus, for further biosorption at 25 ℃ for 36 ~ 48 h in incubator. The electrostatic assembly with transitional metallic Cu, Ni, and Co ions were accomplished by using 0.1 mol L^−1^ C_4_H_6_CuO_4_, C_4_H_6_NiO_4_ and C_4_H_6_CoO_4_ solutions for 24 h. Then, the obtained aerogels were pretreated with liquid nitrogen for 10-min before a freeze drying process. The final hybrid composites were obtained by two-step calcinations, specifically, pyrolysis and gas phase selenylation processes in Ar atmosphere at 500 ℃ for 2 h in a tube furnace, the dosage of Se powder during selenization conversion is 1.0 g.

### Characterization and Measurements

For the composite of hybrid aerogels: SEM and TEM images were investigated through a field emission scanning electron (Magellan 400) and transmission electron microscopies (Tecnai G2 F20 S-TWIN 200KV). BET was obtained by specific surface area analyzer (JW-BK132F). Zeta potential, TG-DSC, XRD, FTIR, Raman, and XPS were performed through Zetasizer Nano thermogravimetric analyzer (NETZSCH STA449 F5/F3 Jupiter), Japanese diffractometer (RIGAKU, D/MAX 2550 V), infrared spectrometer (Nicolet IS10), X-ray photoelectron spectroscopy (Thermo Scientific Escalab 250 Xi) and Raman spectroscopies (Renishaw inVia). The anodes were obtained after coating on Cu foil operation using slurry containing 80% active materials, 10% Super P and 10% polyvinylidene difluoride (PVDF) in *N*-methylpyrrolidone. We use CR2032 type coin cells, Whatman glass fiber (GF/D) for separators, 1.0 M NaClO_4_ and 0.8 M KPF_6_ in ethylene carbonate (EC)/dimethyl carbonate (DMC) (1:1, v/v) for electrolytes. The half-batteries were assembled in an Argon gas filled glovebox. TG-DSC was conducted at the condition of 25 ~ 1100 ℃ under a heating speed of 10 ℃ min^−1^ in air atmosphere. The cyclic voltammetry (CV) was performed by a CHI760E electrochemical working station. Galvanostatic charge–discharge (GCD), cyclic stabilities, rate capabilities and galvanostatic intermittent titration technique (GITT) were finished by LAND CT2001A systems.

## Results and Discussion

### Hybridization and Physical Characterizations of MSe-MXene-CNRib

**Scheme 1:**
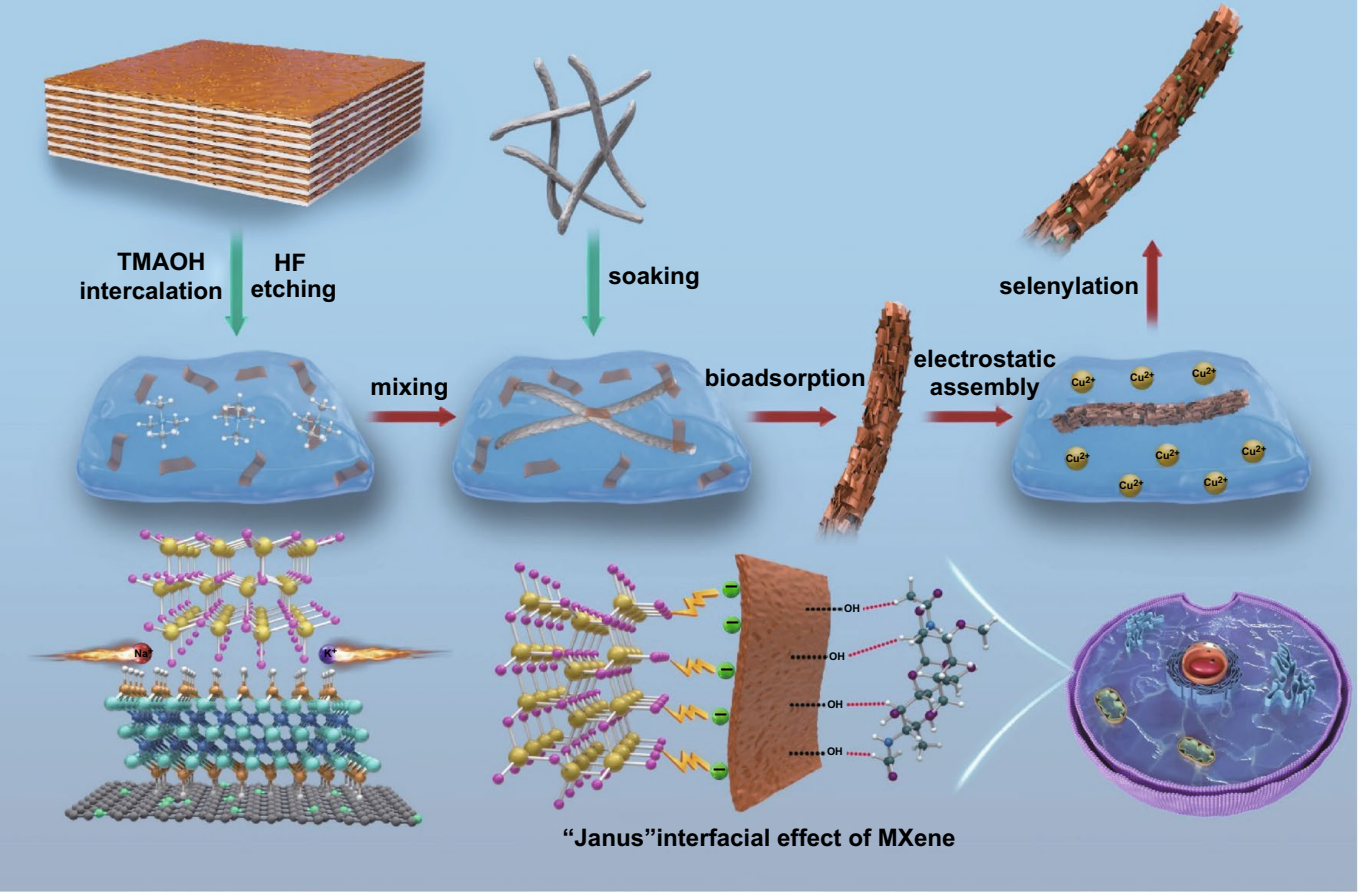
Schematic illustration of transitional metal chalcogenide (TMC) on MXene coated fungal derived carbonaceous nanoribbon (CNRib) heterostructure (TMC@MXene@CNRib)

**Scheme 2:**
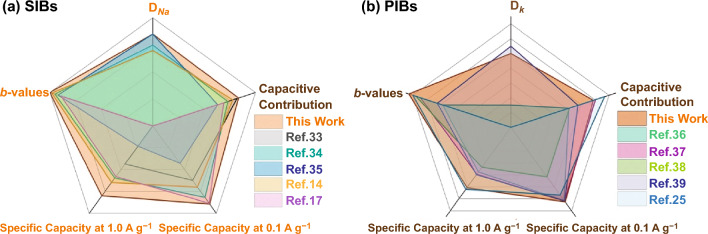
Summary of electrochemical Na/K storages abilities of Cu_1.75_Se-MXene-CNRib ternary heterostructure and similar anodes

As shown in Scheme 1, the fabrication of sandwiched MSe-MXene-CNRib heterostructures was carried out through a two-step self-assembly approach, by which the 2D MXene nanosheets were first assembled onto microbial nanoribbons, *Aspergillus niger*, and then, transition metal ions were deposited onto the surfaces of MXene nanosheets to form sandwiched heterostructures owing to the oppositely charged between ions and Ti_3_C_2_T_*x*_ nanosheets, which can be confirmed by Zeta potentials in Fig. S1. After a calcination and a selenization processes, the freezing dried heterostructured aerogels were converted into TMC-MXene-CNRib heterostructures (Fig. S2). The *Aspergillus niger* used in the fabrication is a low-cost and bio-safe chitin-based microfungus in the form of nanoribbons and is full of hydroxide and amino groups on the surfaces, which are chemical affinity towards the MXene nanosheets terminated with hydroxide groups. After calcination, this microfungus can be easily converted into N-rich carbonaceous nanoribbons with abundant electrochemically active sites and excellent electric conductivity Scheme 2.

**Fig. 1:**
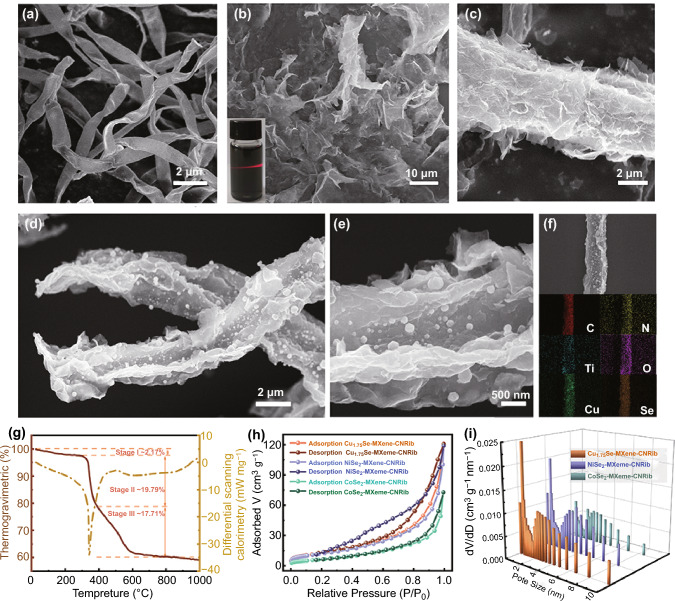
Morphological characterization of precursor materials and the heterostructure composed of transitional metal selenide, Ti_3_C_2_T_*x*_ MXene, and fungus-derived N-rich carbonaceous nanoribbons (MSe-MXene-CNRib): **a** SEM image of *Aspergillus niger* derived carbonaceous nanoribbons. **b** SEM image of few-layered Ti_3_C_2_T_*x*_ MXenes. **c** SEM image of MXene@fungus derived carbonaceous nanoribbon. **d** SEM image and **e** magnified image of Cu_1.75_Se-MXene-CNRib. **f** STEM elemental mapping of Cu_1.75_Se-MXene-CNRib. **g** TG-DSC profile of Cu_1.75_Se-MXene-CNRib. **h** Nitrogen adsorption–desorption isotherms and **i** pore size distributions of Cu_1.75_Se-MXene-CNRib, NiSe_2_-MXene-CNRib and CoSe_2_-MXene-CNRib

Figure 1 presents the morphologies of the precursors and the assembled MSe-MXene-CNRib heterostructures. Figure 1a shows the SEM image of the dispersed *Aspergillus niger* nanoribbons, which are 12–15 μm in length, around 600 nm in width, and 133 nm in thickness. The *Aspergillus niger* nanoribbons before assembly and pyrolysis have smooth surfaces. The multilayered Ti_3_C_2_T_*x*_ MXene nanosheets were prepared through a widely employed protocol with HF etching (Fig. S3) following by a tetramethylammonium hydroxide (TMAOH) assisted exfoliation step. Figure 1b presents the morphology of the exfoliated Ti_3_C_2_T_*x*_ MXene nanosheets, which can be well-dispersed in water as confirmed by an obvious Tyndall effect (inset in Fig. 1b). Through the hydrogen and amino-bridging bonds formed between the hydroxy terminated (–OH) Ti_3_C_2_T_*x*_ and the hydroxide and amino groups on the chitin in fungus, the MXene nanosheets closely attached on the surfaces of microorganisms via a spontaneous bio-adsorption effect. The formed MXene@fungus CNRibs heterostructure was displayed in Fig. 1c, where the homogenous attachment of Ti_3_C_2_T_*x*_ nanosheets on fungus NRibs in a vertical way was clearly observed. This alignment mode can provide adequate active surface area and active sites for efficient electrochemical applications. To address the challenges existing in MXenes, transition metal selenides (MSe, M = Cu, Ni, and Co) nanoparticles were further grown uniformly on the surfaces of Ti_3_C_2_T_*x*_ nanosheets via a chemical adsorption followed with a selenylation process (Fig. 1d). In brief, the as prepared MXene@fungus NRibs were dispersed into 0.1 mol L^−1^ C_4_H_6_CuO_4_, C_4_H_6_NiO_4_, and C_4_H_6_CoO_4_ solutions, respectively, for 24 h, and then were freezing dried at -55 ℃, and finally calcinated at 500 ℃ for 2 h in Ar with Se powders at the gas inlet end for form MSe-MXene-CNRibs heterostructures. Figure 1d and e present both the low and high magnification morphology of one typical heterostructure, Cu_1.75_Se-MXene-CNRib. It is clear that, the Cu_1.75_Se nanoparticles are homogenously deposited on the surfaces of MXene nanosheets. The particle size of the obtained Cu_1.75_Se varied over a few nanometers to tens of nanometers in diameter. The morphologies of NiSe_2_-MXene-CNRib and CoSe_2_-MXene-CNRib have also been observed under SEM (Figs. S4, S5). Probably owing to the difference in surface chemistry, such as electronegativity and binding energies, the deposition of NiSe_2_ and CoSe_2_ resulted in restacking of the MXene, which will affect the electrochemical applications. The elemental distribution mapping (Figs. 1f and S4, S5) demonstrated that the transition metal selenides nanoparticles decorated onto the surfaces of the MXene nanosheets homogenously.

The weight percentages of each constituents of the MSe-MXene-CNRib aerogels were evaluated by a thermogravimetric-differential scanning calorimetry (TG-DSC) test in Air. Figure 1g shows the typical weight change curves of Cu_1.75_Se-MXene-CNRib by heating to 1000 ℃, the first weight loss region (Stage I: ~ 2.17%) occurred during 100 to ~ 200 ℃, which should be caused by oxidative process of Ti_3_C_2_T_*x*_ and the release of physically absorbed water and residual acids. It can also be proved by the vibration of hydroxyl in subsequent FTIR analysis. In stage II, a sharp decrease in ~ 19.79% over 200–400 ℃ was observed, which should be resulted by the sublimation of SeO_2_ as the oxidation of Cu_1.75_Se nanoparticles into CuO and SeO_2_. The weight change of ~ 17.71% above 400 ℃ (Stage III) can be assigned to the oxidation of CNRibs. The corresponding chemical reactions at each stage thus should occur as follows:

Stage I 100 ~ 200 ℃: Ti_3_C_2_ + 5 O_2_ → 3 TiO_2_ + 2 CO_2_ ↑

Stage II 200 ~ 400 ℃: 8 Cu_1.75_Se + 15 O_2_ → 14 CuO + 8 SeO_2_ ↑

Stage III Above 400 ℃: C + O_2_ → CO_2_ ↑

Based on the weight loss and the chemical reactions, the composition of the ternary Cu_1.75_Se-MXene-CNRib heterostructure fabricated in this case was estimated as 79.1% Cu_1.75_Se, 9.6% MXene, and 11.3% CNRib. The compositions of NiSe_2_-MXene-CNRib and CoSe_2_-MXene-CNRib were also evaluated in the same way (Figs. S6, S7).

Brunauer Emmett Teller (BET) examination was employed to investigate textural properties of the MSe-MXene-CNRib heterostructures (Fig. 1h), where typical *type III* isotherms with H3 hysteresis loops were observed in all samples, showing the co-existence of disordered meso- and microporous structures. The BET surface areas of three aerogels were calculated as 77.82, 66.23, and 59.28 m^2^ g^−1^, respectively, for Cu_1.75_Se-MXene-CNRib, NiSe_2_-MXene-CNRib, and CoSe_2_-MXene-CNRib, as well as the average pore sizes of 10.38, 6.67, and 7.15 nm (Fig. 1i). The high specific surface area and the existence of appropriate micro/mesopores are favorable of providing sufficient accessible interfaces between electrode/electrolyte for ions diffusion and extra mass transport channels in the targeted electrochemical applications.

**Fig. 2:**
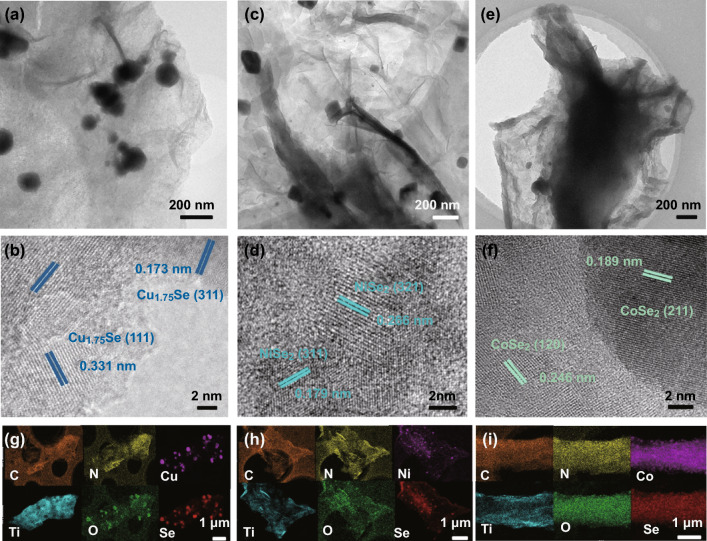
Structural characterization of precursor materials and the heterostructure composed of transitional metal selenide, Ti_3_C_2_T_*x*_ MXene, and fungus-derived N-rich carbonaceous nanoribbons (MSe-MXene-CNRib): TEM and HRTEM images for **a–b** Cu_1.75_Se-MXene-CNRib, **c–d** NiSe_2_-MXene-CNRib, and **e–f** CoSe_2_-MXene-CNRib. **g–i** Elemental mappings for Cu_1.75_Se-MXene-CNRib, NiSe_2_-MXene-CNRib, and CoSe_2_-MXene-CNRib

To give insight into the detailed crystal structures of the MSe-MXene-CNRib, the microstructures were further characterized by transmission electron microscopy (TEM) technique (Fig. 2). The low magnification bright-field images (Fig. 2a, c and e) clearly illustrate the dispersed nanosheet structure of Ti_3_C_2_T_*x*_ MXene and the evenly adsorbed MSe nanoparticles on the MXene. The high-resolution TEM (HRTEM) images, as presented in Fig. 2b, d, and f, give the detailed crystal structure of MSe@MXene. The lattice fringe images of MSe and TiO_2_ resulted by the partial oxidation of MXene were clearly identified, as indicated by the mazarine-colored dotted lines. In these HRTEM images, the Cu_1.75_Se nanoparticles exposed with (111), (220), and (311) facets and TiO_2_ exposed with (400) (Fig. 2b) facet were identified in the Cu_1.75_Se-MXene-CNRib heterostructure, and fringes corresponding to the (311) and (210) planes of NiSe_2_ and the (221), (211), and (120) planes of CoSe_2_ were also clearly observed in NiSe_2_-MXene-CNRib (Fig. 2d) and CoSe_2_-MXene-CNRib (Fig. 2f), respectively. Table S1 lists the estimated chemical composition of the fabricated heterostructures based on the EDX data (Fig. S8). Figure 2g–i presents the elemental distributions of each heterostructure, which confirmed the distribution of C, N, and O elements in the carbonaceous nanoribbons and MXene nanosheets and the Cu, Ni, Co, and Se elements in the outer chalcogenides.

**Fig. 3:**
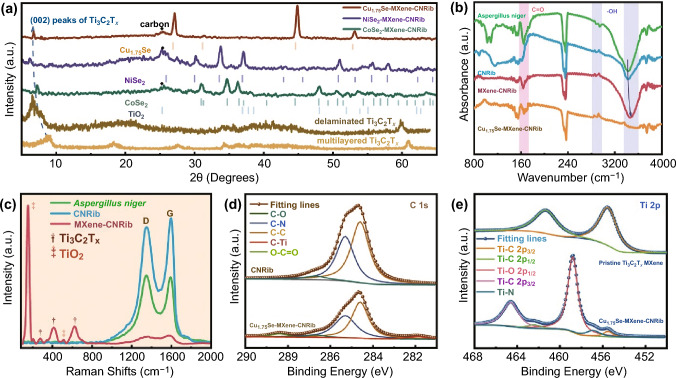
Compositional analysis of precursor materials and the heterostructure composed of transitional metal selenide, Ti_3_C_2_T_*x*_ MXene, and fungus-derived N-rich carbonaceous nanoribbons (MSe-MXene-CNRib): **a** XRD patterns of Ti_3_C_2_T_*x*_, Cu_1.75_Se-MXene-CNRib, NiSe_2_-MXene-CNRib and CoSe_2_-MXene-CNRib. **b** FTIR results of *Aspergillus niger*, CNRib, MXene-CNRib and Cu_1.75_Se-MXene-CNRib. **c** Raman shifts of *Aspergillus niger*, CNRib and MXene-CNRib. **d** High-Resolution C 1 s XPS spectra of CNRib and Cu_1.75_Se-MXene-CNRib. **e** High-Resolution Ti 2p XPS spectra of pristine Ti_3_C_2_T_*x*_ MXene and Cu_1.75_Se-MXene-CNRib

To investigate the bonding behaviors and chemical states of the MSe-MXene-CNRib heterostructures, various spectroscopy techniques have been employed. Figure 3a displays the X-ray diffraction (XRD) patterns of the as-etched Ti_3_C_2_T_*x*_, the as-exfoliated Ti_3_C_2_T_*x*_ nanosheets, and the assembled MSe-MXene-CNRib heterostructures. The characteristic peak of (002) plane for Ti_3_C_2_T_*x*_ shifted from 8.9° to 6.5° during liquid exfoliation, indicating the effective TMAOH intercalation into MXenes and the increase in interlamellar spacing to 1.3 nm. After assembly and selenylation into MSe-MXene-CNRib, this (002) plane showed slight variations, i.e., 6.7°, 6.1°, and 7.3°, respectively, for Cu_1.75_Se-MXene-NCRib, NiSe_2_-MXene- CNRib, and CoSe_2_-MXene-CNRib, confirming the well-dispersion of the MXene nanosheets onto the 1D carbonaceous nanoribbons. The existence of anatase TiO_2_ was also detected, owing to the slight oxidization of Ti_3_C_2_T_*x*_ MXene during the selenylation process. The Fourier transform infrared (FT-IR) stretching vibration modes in Cu_1.75_Se-MXene-CNRib (Fig. 3b) provide insights into the bonds formed between the constituents of the heterostructure, where the vibration peaks located at 3420 cm^−1^ shifting upwards to higher wavelength of 3477 cm^−1^, might contributed by the formation of hydrogen bonds between the terminal hydroxyls on Ti_3_C_2_(OH)_2_ and the chitin in fungal cell wall [18]. Particularly, after pyrolysis, the -OH in chitin were well retained, guaranteeing the high affinity between MXenes and fungus by providing adequate active sites for hydrogen bonds generation [19, 20]. Furthermore, the adsorption peaks at around 1651 and 1708 cm^−1^ can be classified as the bond of C=O in both Ti_3_C_2_O_2_ and biofungi, which is favorable for boosting electrochemical storages performance [21]. From the Raman spectra variation in Fig. 3c, we observed that after pyrolysis process of biofungus, the graphitic vibrations ratio of D and G modes reached as high as 0.93, suggesting the high graphitic degree of CNRib with fast electronic conductivity. Additionally, the characteristic peaks of Ti_3_C_2_T_*x*_ and its surficial oxidative TiO_2_ appeared in Raman spectrum after MXene bonding via stable hydrogen bonds, indicating the strong coupling between the 2D MXene nanosheets and the biofungal carbon nanoribbons, which offers possibility for ultrastable post-Li ions storages.

To further investigate the bonding behaviors at the MXenes “Janus” interfaces, X-ray photoelectron spectroscopy (XPS) was performed to evaluate the chemical states of ternary heterostructure. By feat of the hydrogen bonds formation, in the C 1 s spectra of Cu_1.75_Se-MXene-CNRib (Fig. 3d), an emerging peak situated at around 282.2 eV can be assigned to C-Ti associated with the synergistical assembly, indicating the stable bonding behaviors between the Ti_3_C_2_T_*x*_ and the carbonaceous nanoribbons [22, 23]. Compared to pristine Ti_3_C_2_T_*x*_, the appearance of Ti–O in Ti 2p spectra of ternary nanohybrids demonstrates the partial oxidation process for surficial Ti atoms during hybridization. Furthermore, a noticeable peak located at about 456.9 eV can be ascribed to the Ti-N resulted by the adsorption of MXene and N-rich carbonaceous fibers, suggesting the effective bio-adsorption between the *Aspergillus niger* fibers and the MXene nanosheets as well as the chalcogenide decorations [24].

It can be clearly observed that two distinguishable peaks in Cu 2p were situated at around 932.3 and 952.2 eV, which can be attributed to Cu 2p_1/2_ and Cu 2p_3/2_ of Cu_1.75_Se, respectively, in Fig. S10a, indicative of valence state closing to +1. Meanwhile, the Se 3d spectra in Fig. S10b could be classified to three specific signals centered at about 54.1, 55.2, and 59.6 eV, assigning to the electronic orbits of 3d_5/2_ and 3d_3/2_ of Se^2−^, as well as the Se-Se or Se-O bonds resulting from the selenylation procedure [21]. As for NiSe_2_-MXene-CNRib and CoSe_2_-MXene-CNRib aerogels, the corresponding high-resolution Ni 2p and Co 2p spectra are explicitly displayed in Fig. S11, and similar chemical states with those of Cu_1.75_Se-MXene-CNRib have been identified [25, 26].

Based on the above microstructure and surface chemistry analysis, as we expected, strongly coupled MSe-MXene-CNRib heterostructures have been successfully fabricated. This type of novel heterostructures features vertically aligned 2D MXene nanosheets with open structures, electric conductive 1D carbonaceous nanoribbon networks, homogeneous TMC nanoparticle surface decoration on MXene nanosheets, strongly coupled interfaces between the TMC/MXene and the MXene/CNRibs. We expected that this unique ternary heterostructures could offer extraordinary electrochemical activity and performance towards sustainable energy storages, particularly for those suffering from kinetically sluggish large alkali ions. As proof-of-concepts, we assembled the innovated heterostructure as the anode in rechargeable batteries to host Na and K ions with large radii and evaluated their performance and potential for high-rate energy storage in SIBs and PIBs.

### Electrochemical Properties Evaluation of MSe-MXene-CNRib in SIBs

**Fig. 4:**
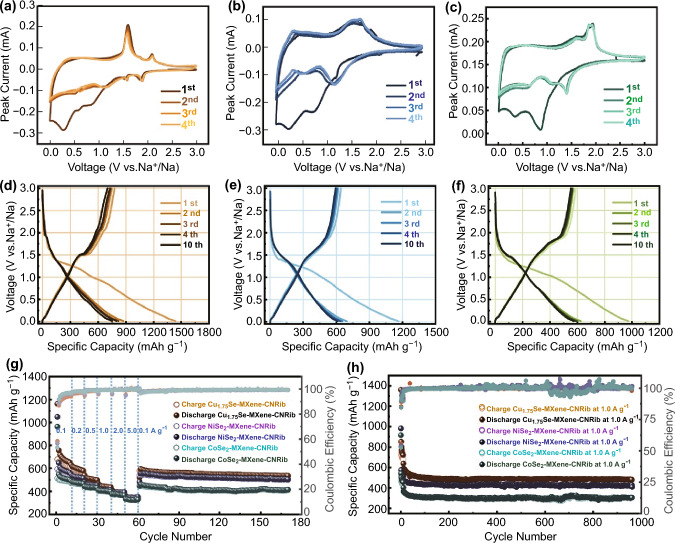
Electrochemical Na storage of the MSe-MXene-CNRib heterostructures: CV curves for first three cycles at the sweep rate of 0.2 mV s^−1^ for **a** Cu_1.75_Se-MXene-CNRib, **b** NiSe_2_-MXene-CNRib, and **c** CoSe_2_-MXene-CNRib. GCD profiles for some specific charge/discharge cycles for **d** Cu_1.75_Se-MXene-CNRib, **e** NiSe_2_-MXene-CNRib, and **f** CoSe_2_-MXene-CNRib. **g** Rate capability and cycling stabilities at 0.1 A g^−1^. **h** Cycling stabilities at 1.0 A g^−1^

Figure 4 illustrates the electrochemical performance of strongly coupled MSe-MXene-CNRib heterostructures for Na-ion storage as the anode in a SIB. Figure 4a–c shows the initial three cyclic voltammetry (CV) curves, respectively, for Cu_1.75_Se-MXene-CNRib, NiSe_2_-MXene-CNRib, and CoSe_2_-MXene-CNRib at a sweep rate of 0.2 mV s^−1^. For Cu_1.75_Se-MXene-CNRib (Fig. 4a), the irreversible peaks in the first scan can be ascribed to the solid-electrolyte-interfaces (SEI) films formation resulted by the insertion of sodium ions into the hybrid materials, as indicated by the appearing redox peaks at around 1.9 and 1.1 V. The following two peaks in the range of 0.68–0.35 V should correspond to the generation of NaCu_1.75_Se during the alloying reaction and the further decomposition into Cu and Na_2_Se. In the anodic scan, the intensive peak at ~ 1.54 V should be the recovery of NaCu_1.75_Se alloy with sodiation, followed by a noticeable redox peak at ~ 2.05 V corresponding to the Cu_1.75_Se reformation via the desodiation reactions. The succeeding sweep profiles, however, presented satisfactory reversibility during the consecutive insertion and extraction of Na^+^, even though an irreversible capacity loss resulted from the formation of SEI was also identified. Similar electrochemical redox reactions were observed for the heterostructures with NiSe_2_ (Fig. 4b) and CoSe_2_ (Fig. 4c). According to the monitored Na-ion insertion and extraction process, the electrode redox reactions for each heterostructure can be proposed as follows [27, 28, 29]:

1 $${\text{Cu}}_{1.75} {\text{Se}}\overset{{{\text{Na}}^{ + } + {\text{e}}^{ - } }} \longleftrightarrow {\text{Na}}_{x} {\text{Cu}}_{1.75} {\text{Se}}\overset{{{\text{Na}}^{ + } + {\text{e}}^{ - } }}\longleftrightarrow {\text{ Cu}} + {\text{Na}}_{2} {\text{Se}}$$

2 $${\text{NiSe}}_{2} \overset {{\text{Na}}^{ + } + {\text{e}}^{ - } } \longleftrightarrow {\text{Na}}_{y} {\text{NiSe}}_{2} \overset {{\text{Na}}^{ + } + {\text{e}}^{ - } } \longleftrightarrow {\text{Ni}} + {\text{Na}}_{2} {\text{Se}}$$

3 $${\text{CoSe}}_{2} \overset {{\text{Na}}^{ + } + {\text{e}}^{ - } } \longleftrightarrow {\text{Na}}_{z} {\text{CoSe}}_{2} \overset {{\text{Na}}^{ + } + {\text{e}}^{ - } } \longleftrightarrow {\text{CoSe}} + {\text{Na}}_{2} {\text{Se}}\overset {{\text{Na}}^{ + } + {\text{e}}^{ - } } \longleftrightarrow {\text{Co}} + {\text{Na}}_{2} {\text{Se}}$$

Besides the redox reactions assigned to the insertion/deinsertion of Na ions of MSe, the MXene also contributed to the Na-ion storage. As we mentioned above, some Ti atoms within Ti_3_C_2_T_*x*_ MXenes were partially oxidized into TiO_2_ during annealing and vapor-phase selenizing process. A pair of nonnegligible redox peaks situated at ~ 0.6 and ~ 1.5 V appeared in the CV scans, which should be a consequence of the transport of Na ions into or out of the TiO_2_@Ti_3_C_2_T_*x*_ interfaces. Besides that the quasi-rectangle outline of CV curves at low potential range of 0.01 ~ 1.25 V can be ascribed to the intercalation and pseudocapacitive Na-ion storages of the MXene.

4 $${\text{Ti}}_{3}{\text{C}}_{2}{\text{T}}_{x}\overset {{\text{Na}}^{ + } + {\text{e}}^{ - } } \longleftrightarrow {\text{Na}}_{i}{\text{Ti}}_{3}{\text{C}}_{2}{\text{T}}_{x}$$

5 $${\text{TiO}}_{2} \overset {{\text{Na}}^{ + } + {\text{e}}^{ - } } \longleftrightarrow {\text{Na}}_{{\text{j}}} {\text{TiO}}_{2}$$

The synergetic storages contributed by different active components and the various interfaces resulted in a multi-stage storage behavior and thus can dramatically enhance the storage performance of the electrode.

We would not forget to mention both the mechanical and electric integrity contributed by the highly conductive N-rich carbonaceous networks, which not only maintain the structural stability and alleviate the self-restack of the 2D nanomaterials, but also provide a rapid charge transport networks and enhance the electrochemical stability of the electrode. The galvanostatic charge/discharge (GCD) profiles for the first four and the tenth cycles of the Cu_1.75_Se-MXene-CNRib, NiSe_2_-MXene-CNRib, and CoSe_2_-MXene-CNRib anodes at 0.1 A g^−1^ are illustrated in Fig. 4d–f. Besides the confirmation of a multistage conversion reaction mechanism by the multiple potential-capacity plateaus, highly reversible and stable charging-discharging of Na ions after the 1^st^ cycle were recorded.

Figure 4g shows the rate capabilities of each anode followed with a cyclic stability measurement at a small current density of 0.1 A g^−1^. The Cu_1.75_Se-MXene-CNRib anode delivered an ultrahigh initial discharge capacity of 1162.4 mAh g^−1^ at 0.1 A g^−1^, but dropped to 678.6 mAh g^−1^ for charging, resulting in a relatively low initial coulombic efficiency (ICE) of 58.37%, indicating irreversible consumption of Na for the SEI formation and incomplete dealloying. After the first few cycles, the reversible specific capacities maintained at 611.2, 519.5, 451.0, 392.8, and 355.3 mAh g^−1^, respectively, with the stepwise increase of current density from 0.1 to 5.0 A g^−1^. Particularly, the retained reversible capacity was stabilized at 536.3 mAh g^−1^ at 0.1 A g^−1^ up to 100 cycles, together with excellent CEs of ~ 99.43%. Owing to the similar composition and structure, the NiSe_2_-MXene-CNRib and CoSe_2_-MXene-CNRib anodes presents very similar storage and cycling performance, except for lower storage capacity toward Na-ion, the eversible capacity of NiSe_2_-MXene-CNRib was 504.9 mAh g^−1^ at 0.1 A g^−1^ with CEs of 99.2%, and it was 415.3 mAh g^−1^ at 0.1 A g^−1^ with CEs close to 99.4% for CoSe_2_-MXene-CNRib. In addition, the EIS results for all MSe-MXene-CNRib are illustrated in Fig. S12, the sharp drop of semicircles in the region of high to middle frequency indicates the dramatical decrease in charge transfer resistance during Na ions repeating insertion/deinsertion cycling, resulting in the enhanced conductivity of heterostructure to deliver better rate capabilities [30].

For the storage of kinetic-sluggish Na^+^ and K^+^, we are more interested in the performance at higher rates. Figure 4h presents the cyclic performance at 1.0 A g^−1^ up to 1000 cycles. It is very applausive that the reversible capacities of the heterosturctured anodes maintained at very high values without obvious decay even cycled to 1000 cycles, which were 480.7, 416.1, and 302.4 mAh g^−1^, respectively, for Cu_1.75_Se-MXene-CNRib, NiSe_2_-MXene-CNRib, and CoSe_2_-MXene-CNRib, with CE of 90% in the first four cycles and then around 99.4–99.6% in the thereafter repeating alloying/dealloying cycles. The average capacity decay was calculated as 0.37 mAh g^−1^ (0.0017%), 0.16 mAh g^−1^ (0.0007%), and 0.24 mAh g^−1^ (0.0005%) per cycle, respectively, for the Cu_1.75_Se-MXene-CNRib, NiSe_2_-MXene-CNRib, and CoSe_2_-MXene-CNRib anodes. It is thus very clear that the design of this type of strongly coupled ternary heterostructures can offer ultrahigh cycling reliability and reversibility for large Na ions at both low and high rates over 1,000 cycles.

### Ultrafast Ions Transportation Kinetic Analysis of MSe-MXene CNRib in SIBs

**Fig. 5:**
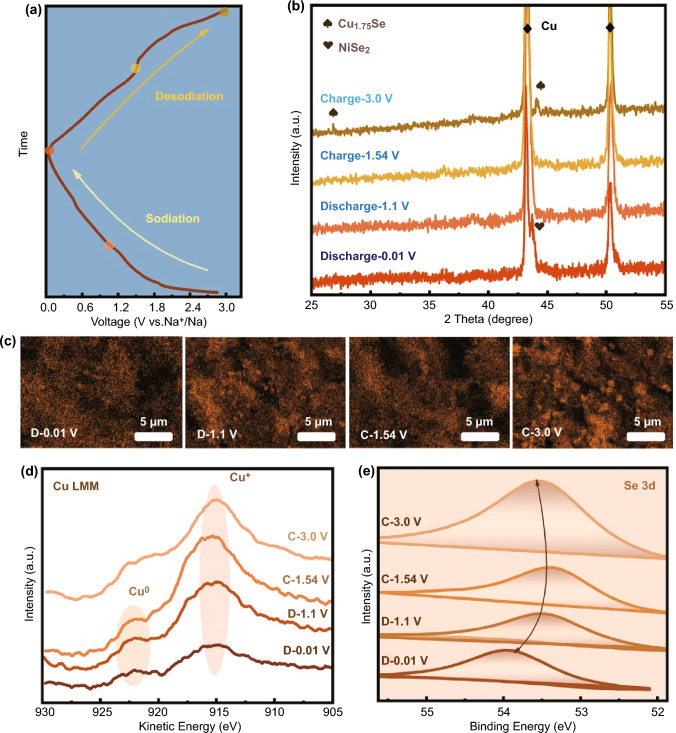
**a** Discharging-charging profiles of Cu_1.75_Se-MXene-CNRib anode for Na storage. **b** *ex-situ* XRD patterns of Cu_1.75_Se-MXene-CNRib anode. **c** STEM Na elemental mappings of Cu_1.75_Se-MXene-CNRib anode at different states. **d** *ex-situ* Cu LMM and **e** Se 3d core spectra at different charging/discharging stages

**Fig. 6:**
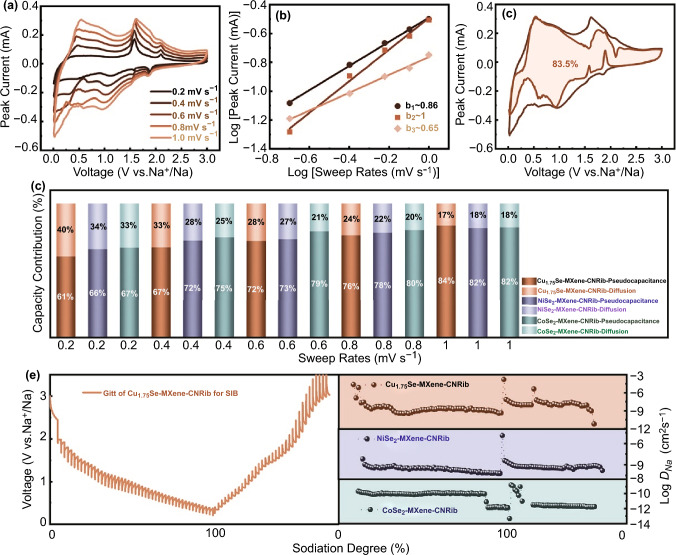
Na^+^ dynamic properties based on the electrochemical measurement of the heterostructure composed of transitional metal selenide and fungus-derived N-rich carbonaceous nanoribbons (MSe-MXene-CNRib): **a** CV curves at different sweep rates for Cu_1.75_Se-MXene-CNRib. **b** Linear logarithmic relationships between peak current *vs.* various sweep rates for Cu_1.75_Se-MXene-CNRib. **c** CV curves at 1.0 mV s^−1^ with the shaded area refers to the pseudocapacitive-dominated proportion for Cu_1.75_Se-MXene-CNRib. **d** Normalized capacity contributions ratios for both diffusion-controlled and pseudocapacitive process of MSe-MXene-CNRib nanohybrids at various sweep rates. **e** GITT potential profiles for Cu_1.75_Se-MXene-CNRib and corresponding Na ions diffusivities *vs.* states of sodiation/desodiation for MSe-MXene-CNRib samples

To understand the kinetic process of the Na-ion insertion and extraction, *ex-situ* XRD measurements, rate varied CV scanning, galvanostatic intermittent titration techniques (GITT), were employed to identify the phase evolution during the alloying-dealloying conversion reactions at different stages (Figs. 5, 6). Figure 5b show the XRD patterns of Cu_1.75_Se-MXene-CNRib anode at four specific potential points during the discharge/charge cycle with corresponding stages in Fig. 5a. At the state of fully charged at 3.0 V, two peaks of Cu_1.75_Se were found at 26.8° and 44.6° (JCPDS No.05-4915), indicating the existence of Cu_1.75_Se. When anode discharged to 0.01 V, the signal of Na_2_Se at 44.1° was observed, suggesting the completion of Na^+^ intercalation, even though the characteristic peak for the metallic Cu decomposed from Cu_1.75_Se was overlapped with peaks for the copper foil current collector. As for an intermediate state of charged at 1.54 V and discharged at 1.1 V, no intermediate phase, such as Na_x_Cu_1.75_Se, can be identified, owing to its low crystallinity. Figure 5c presents the STEM Na element mapping of the corresponding anodes. We can see that there is an obvious tendency of Na aggregation at relative high potentials points during desodiation process, revealing the alloying/dealloying evolution between Na^+^ and ternary Cu_1.75_Se-MXene-CNRib heterostructure hybrids. The co-presence of Cu elemental various valences could be further investigated through *ex-situ* X-ray induced auger electron spectroscopy (XAES) spectra of Cu LMM at different Na^+^ insertion/extraction states (Fig. 5d), the Cu LMM Auger peak situated at around 915.4 eV (kinetic energy) can be assigned to the cuprous existence in ternary hybrids. Over Na ions insertion continues, the appearance of auger peak at around 921.8 eV refers to the zerovalent Cu, illustrating the generation of Cu at lower potentials during electrochemical alloying and conversion reactions, which is in accordance with the evolution analysis based on the ex-situ XRD patterns. Given the formation of polyselenides during charging/discharging process, the Se 3d spectra shifts back to higher binding energy at the fully sodiation state in Fig. 5e, suggesting the final conversion of polyselenides, presumedly Na_2_Se [14, 31].

Dynamic electrochemical responses of the heterostructured electrode can be examined by CV scanning at different scan rates. In theory, from the rate-varied CV scans, the capacity contributions can be distinguished qualitatively into two main components, according to a logarithmic relationship between the peak current *i* and the voltage *v* as follows [32]:

6 $${\text{log}}i = {\text{ log }}a \, + \, b{\text{ log}}v$$

The first part is the Faradic storage contributed from the diffusion-driven and pseudocapacitive processes, and the second part is the non-Faradic from the denied electrical double-layered capacitance. If the *b* value close to 1.0, the charge storage mechanism can be considered as surface-controlled storage, otherwise will be a diffusive controlled process. Figure 6a displays the CV scans of Cu_1.75_Se-MXene-CNRib from 0.2 to 1.0 mV s^−1^, where three discernible redox peaks locating at ~ 1.0, ~ 0.5, and ~ 1.6 V were clearly observed at all profiles. According to Eq. (6), the *b* values for these three peaks were deviated from the slop of log *i* versus log *v* (Fig. 6b). For the anodic peak at around 1.0 V, *b* = 0.65 was obtained, which is close to 0.5 and can be assigned to Na^+^ intercalation. For the cathodic peaks at 0.52 and 1.6 V, the b values were 0.99 and 0.86, indicating the ions extraction processes. The varied b values indicate that a hybrid mechanism with both diffusion-dominated and surface pseudocapacitive processes works for the Na-storage of the ternary heterostructure anodes. Further quantitively calculation in the light of a relationship of *i* current response at a fixed voltage *V* can deduce the disparate contributions proportions, in which *i(V)* is the summation of *k*_*1*_*v* and *k*_*2*_*v*^0.5^ (*v* is the scan rates), namely the pseudocapacitive and diffusion-driven contributions, and k_1_ can be calculated from the slope of peak currents *vs.* square roots of sweep rates (Figs. S13–S15). Based on this relationship, as shown in Fig. 6c, the surface capacitive contribution was up to 83.5% of whole capacity at the sweep rate of 1.0 mV s^−1^. Such a high proportion of pseudocapacitive contribution allows rapid storage and transport of Na ions and offers satisfactory rate capabilities at various current densities of the Cu_1.75_Se-MXene-CNRib during the long-term repeating Na^+^ alloying and dealloying operations. Similar storage behaviors have also presented in the case of NiSe_2_-MXene-CNRib (Fig. S16) and CoSe_2_-MXene-CNRib (Fig. S17). The pseudocapacitive storage contributed to 81.7% and 82.3% at 1.0 mV s^−1^, respectively, for NiSe_2_-MXene-CNRib and CoSe_2_-MXene-CNRib anodes. Figure 6d summaries pseudocapacitive contributive fractions at remaining sweep rates as 0.2, 0.4, 0.6, and 0.8 mV s^−1^. In detail, the surface-redox reactions accounted for 61%, 67%, 72%, and 76% for Cu_1.75_Se-MXene-CNRib, 66%–78% for NiSe_2_-MXene-CNRib, and 67%–80% for CoSe_2_-MXene-CNRib. We can see a moderate growth of pseudocapacitive proportion as the increment of the sweep rates.

From the perspective of insertion and extraction of sodium ions, we evaluated the diffusion properties of Na ions during electrochemical reversible redox reactions by virtue of galvanostatic intermittent titration techniques (GITT) (Fig. 6e) [33]. Over the operating potential range, the overpotentials of the heterostuctured anodes were observed a gradual decline tendency as the deepening of sodiation. Vice versa, the overpotentials increased moderately as the ongoing of desodiation. As presented in Figs. 6e and S18, the overvoltage distinction became more evident at relatively high potential responses. In view of sodium ions diffusion efficiency, the diffusion coefficient *D*_*Na*_ were evaluated according to the GITT profiles over the whole sodiated/desodiated processes. Similar variation tendency can be seen in all MSe-MXene-CNRib samples, as deepening of the insertion of Na^+^, the *D*_*Na*_ decreased progressively. At the time of de-embedding of Na^+^ started at the specific potential, the *D*_*Na*_ rocketed to a high value, which might be for a result of abundant effective chemical adsorption sites on the surface and edges of the heterostructures for Na^+^ storage. With the profound storage in the interlayered spaces of the active materials and the deeper interfaces between the MXene nanosheets and the conductive N-doped carbonaceous fibers, the migration of Na^+^ must overcomes higher electrostatic repulsive force and diffusion energy barriers, leading to the drop of *D*_*Na*_ (right plot of Fig. 6e). Coincided with the capacity profiles, the *D*_*Na*_ of Cu_1.75_Se-MXene-CNRib was in a range of 10^–8^ to 10^–9^, which is larger than that of NiSe_2_-MXene-CNRib (~ 10^–9^) and CoSe_2_-MXene-CNRib (~ 10^–10^ to 10^–12^). It means that the sodium ions are capable of more rapid diffusing behaviors in the Cu_1.75_Se-MXene-CNRib heterostructure than others, giving rise to a better rate performance [14, 29, 34, 35, 36].

### Electrochemical Properties Evaluation of Cu_1.75_Se-MXene-CNRib in PIBs

**Fig. 7:**
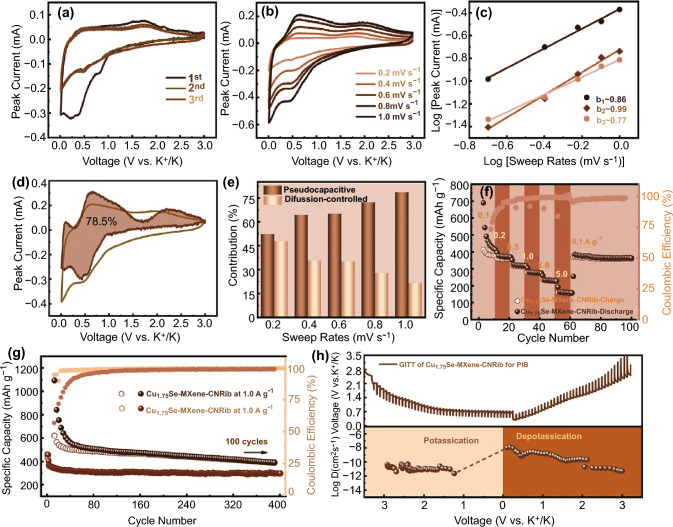
Electrochemical K storage of Cu_1.75_Se-MXene-CNRib heterostructure: **a** CV curves for first three cycles at the sweep rate of 0.2 mV s^−1^. **b** CV curves at various sweep rates. **c** Linear logarithmic relationships between peak current *vs.* various sweep rates. **d** CV curves with dash areas refers to pseudocapacitive-dominated proportions at the sweep rates of 1.0 mV s^−1^. **e** Normalized capacity contributions ratios for both diffusion-controlled and pseudocapacitive process. **f** Rate capabilities, **g** cycling performance at 0.1 and 1.0 A g^−1^. **h** GITT profiles and corresponding *D*_*K*_ values

According to the above electrochemical characterizations on the Na-ion storage performances of MSe-MXene-CNRib, the Cu_1.75_Se-MXene-CNRib heterostructure exhibits a superior cyclic robustness and superb rate properties, enlightening us the potentiality of this promising class of material for acting as anode materials to host other larger alkali ions, such as potassium ions. The K^+^ storage performance of Cu_1.75_Se-MXene-CNRib was thus examined as the anode for PIB batteries. Very similar electrochemical behaviors were observed for the K^+^ storage in Cu_1.75_Se-MXene-CNRib electrode. As shown in Fig. 7a, SEI growth and electrolyte ions decomposition should contribute to the irreversible peaks in the first cycle. The two redox peaks situated at around 0.47 and 0.25 V reflected a consecutive potassium ions insertion and redox conversion progress associated with the generation of K_z_Cu_1.75_Se alloy and followed decomposition into metallic Cu and potassium selenide. In the anodic sweep, the broad peak at around 1.75 V could be assigned to the regeneration of Cu_1.75_Se after an oxidation reaction. The galvanostatic branches monitored at 0.1 A g^−1^ during the consecutive potassiated and depotassiated processes gave further information about the capacity and reversibility of the anode (Fig. S19), where the initial discharge and charge capacities were 1353.1 and 744.2 mAh g^−1^, respectively, corresponding to a relatively low initial CE of 54.9% as a result of the SEI formation was observed for the first cycle. To unravel the K^+^ storage mechanism quantitatively, CV curves scanned at different sweep rates from 0.2 to 1.0 mV s^−1^were recorded (Fig. 7b). The diffusion rate of potassium ions was elucidated from the slope of the above-mentioned relationship of *i* ~ *v*^0.5^ for the redox peaks of the rate-varied CV scans, i.e., the b-values. As shown in Fig. 7c, the *b* values for the redox peaks at 0.5, 0.6, and 2.0 V were 0.90 0.99, and 0.80, respectively, indicating a surface-driven capacitive storage behavior for the entire potassium alloying and dealloying cycles. As visualized in Fig. 7d, the capacitive storage at 1.0 mV s^−1^ (the brown colored area) counted for 78.5% of the whole capacity of the anode. We separated the storage of K^+^ in Cu_1.75_Se-MXene-CNRib electrode at each scan rate (Fig. 7e). It is very clear that, due to the larger ions radius of K^+^ than that of Na^+^, the pseudocapacitive storage contributed to a larger part of capacity for K^+^, verifying the Cu_1.75_Se-MXene-CNRib anode is favorable for surface dominated potassium ions storage, particularly at high rates.

### Ultrafast Ions Transportation Kinetic Analysis of Cu_1.75_Se-MXene-CNRib in PIBs

Figure 7f shows the rate stability of the Cu_1.75_Se-MXene-CNRib anode at current densities varying over 0.1 to 5.0 A g^−1^. The reversible K^+^ storage capacities of the electrode remained as high as 435.3, 356.2, 315.7, 274.3, 232.6, and 161.3 mAh g^−1^ with the increase in cycling rates. When the current density turned back to 0.1 A g^−1^ after cycling at each rate, the reversible specific capacity was retained at 375.8 mAh g^−1^ for an extended 40 cycles. The long-term potassium ions insertion and extraction circulation stability were assessed at the rates of 0.1 and 1.0 A g^−1^ (Fig. 7g). Albeit the low initial CEs resulted from the larger radius and the more sluggish dynamics of K^+^, particularly at a low rate, the Cu_1.75_Se-MXene-CNRib anode still delivered a high reversible K^+^ storage capacity of 401.3 mAh g^−1^ at 0.1 A g^−1^ up to 400 cycles with CEs close to 99%. The capacity at 1.0 A g^−1^ was maintained at 305.6 mAh g^−1^ up to 400 cycles with a much better reversibility and stability and the capacity loss for each cycle was only 0.405 mAh g^−1^. Figure 7h exhibits the GITT profiles for both the potassiation and the depotassiation processes, on the basis of potential-time response plot, and the calculated K^+^ diffusion coefficient *D*_*K*_. The *D*_*K*_ values for the potassiation process varied in range of 10^–10^ to 10^–11^ cm^2^ s^−1^ and were 10^–8^ ~ 10^–10^ cm^2^ s^−1^ for the depotassation process. The high K^+^ diffusion coefficients confirmed that the ternary Cu_1.75_Se-MXene-CNRib anode allows rapid K^+^ migration and storage at a more efficient kinetic behavior, and it can be a competitive anode for PIBs [21, 36–39, 40].

### Theoretical Calculations of MSe-MXene-CNRib Heterostructure

**Fig. 8:**
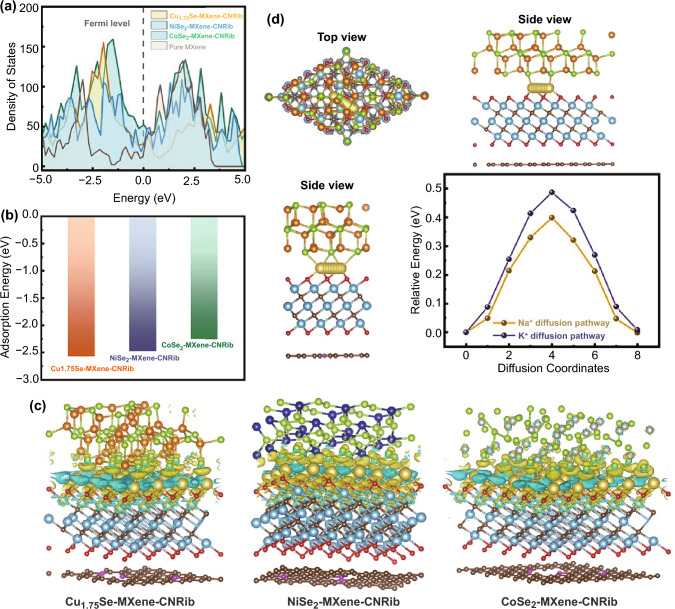
Theoretical calculations of Na/K adsorption and diffusion in the heterostructure composed of transitional metal selenide and fungus-derived N-rich carbonaceous nanoribbons (MSe-MXene-CNRib): **a** Densities of states of Na adsorbed pristine MXene, Cu_1.75_Se-MXene-CNRib, NiSe_2_-MXene-CNRib and CoSe_2_-MXene-CNRib. **b** Calculated adsorption energies of the most stable Na adsorption sites in Cu_1.75_Se-MXene-CNRib, NiSe_2_-MXene-CNRib and CoSe_2_-MXene-CNRib. **c** Charge density differences of Na adsorbed Cu_1.75_Se-MXene-CNRib, NiSe_2_-MXene-CNRib, and CoSe_2_-MXene-CNRib. **d** Energy profiles for Na/K diffusion on the interlayer of Cu_1.75_Se and Ti_3_C_2_O_2_@N-doped carbon

Based on the above electrochemical performance and mechanism analysis, we presume that there is a synergic effect among the ternary MSe-MXene-CNRib heterostructures contributed to the superior large alkali ions storage properties. We thus studied the diffusion behaviors at the interface of the heterostructures by employing the density functional theory (DFT) calculations. The crystal structures used in the calculations can be found in Figs. S20–S22. Figure 8a presents the Densities of states (DOS) of the MSe-MXene-CNRib and the pristine MXene. Notably, all the MSe-MXene-CNRib heterostructures exhibit more electronic states at the Fermi level than that of MXenes, indicating the greatly enhanced electronic conductivities of the heterostructures [41]. Figures 8b and S23 gives the adsorption energy of both Na and K ions on the surfaces of MSe-MXene-CNRib, by which we can know that the Cu_1.75_Se-MXene-CNRib and NiSe_2_-MXene-CNRib are more favorable for Na adsorption and intercalation than CoSe_2_-MXene-CNRib, as well as a stronger Na capture ability. Figure 8c demonstrates the charge distribution difference of the Na adsorbed heterostructures. No significant difference of the charge redistribution patterns among all three heterostructures. Specifically, the Na atoms release the electrons to not only the connected Cu_1.75_Se, NiSe_2_, or CoSe_2_, but also the Ti_3_C_2_O_2_ MXene, which suggests a synergic effect of MSe and MXene on the adsorption of Na atoms [42]. To investigate impact of the adsorption strength on the mobility of alkali ions, the relative energies along the favorable diffusion paths at the interface of Cu_1.75_Se and the MXene/CNRib were investigated, as shown in Fig. 8d. The calculated diffusion barriers for Na and K atoms were 0.3996 and 0.4868 eV, respectively, which are comparable to other anodes with similar materials for SIBs and PIBs. The higher energy barrier for K is mainly attributed to its larger radius and heavier weight. The theoretical calculations gave us the ideas that the existence of proposed synergetic effects between MSe and MXene/NCRib is supposed to promote the Na/K ions adsorption and diffusion kinetics [43].

It is very clear that the novel ternary MSe-MXene-CNRib heterostructures are capable of exceptional Na and K ions storages with excellent capacity at both low and high rates [14, 29, 22, 33–40]. The exceptional electrochemical performance is mainly attributed to the unique combination of 2D nanomaterials, which provide abundant active storage sites on the surfaces and at the interfaces for the large Na and K ions and allow ultrafast ion transport and migration within the porous structures. In summary, the microbe fungus nanofibers offer high chemical affinity toward MXene nanosheets for the vertically growth of MXene on the carbonaceous nanofibers, and the vertically aligned and fully opened MXene nanosheets provide sufficient accessible growth sites for the transitional metal ions adsorption and the formation of MSe, which finally lead to the formation of strongly coupled ternary MSe-MXene-CNRib heterostructures. The highly accessible surfaces and interfaces of the ternary heterostructures provide superb surficial pseudocapacitive storages for both Na and K ions with low energy barriers instead of a sluggish intracrystalline diffusion as demonstrated by both the experimental GITT characterization and the theoretical DFT calculations.

## Conclusion

In our work, a novel class of ternary MSe-MXene-CNRib heterostructures consisting of 2D MXene nanosheets, transition metal selenide nanoparticles, and N-rich carbonaceous nanoribobons with strong interfacial coupling were successfully fabricated through a microbe-assisted self-assembly and a followed selenization process for the application in energy storage devices. These ternary heterostructures possess some unique features: (1) highly conductive inner N-rich carbon nanoribbon networks, (2) vertically aligned MXene nanosheets with fully dispersed open structure, (3) strongly deposited transition metal selenides with abundant active surficial sites and extra storage interfaces, endorsing the hybrid materials for ultrafast interfacial transport of large alkali ions, superb high-rate capacity and ultrastable long-term cycling stability. Taking Cu_1.75_Se-MXene-CNRib heterostructure as an example, the highest reversible Na^+^ storage capacity reached 536.3 mAh g^−1^ @ 0.1 A g^−1^ with Na^+^ diffusion coefficients, *D*_Na_ of 10^–8^–10^–9^ cm^2^ s^−1^, and the stable K^+^ capacity could be 305.6 mAh g^−1^ at 1.0 A g^−1^ after 400 cycles. For the high-rate storage of large Na and K ions, the pseudocapacitive storage contributed dominantly, which allows rapid insertion and de-insertion of the large ions within the electrode. It is thus believed that the strongly coupled 2D-based heterostructures featuring abundant active surface sites, ultrafast interfaces, and highly conductive backbone structures can facilitate the migration and ultrastable storage of large alkali ions and address the current grand challenges in low-cost but kinetically sluggish post-Li-ion batteries.

### Supplementary Information

Below is the link to the electronic supplementary material.

Supplementary file1 (PDF 1630 KB)
