# Publisher Correction to: Strongly Coupled 2D Transition Metal Chalcogenide-MXene-Carbonaceous Nanoribbon Heterostructures with Ultrafast Ion Transport for Boosting Sodium/Potassium Ions Storage

**DOI:** 10.1007/s40820-023-01320-1

**Published:** 2024-02-01

**Authors:** Junming Cao, Junzhi Li, Dongdong Li, Zeyu Yuan, Yuming Zhang, Valerii Shulga, Ziqi Sun, Wei Han

**Affiliations:** 1 Sino-Russian International Joint Laboratory for Clean Energy and Energy Conversion Technology, International Center of Future Science, College of Physics, Jilin University, Changchun, 130012 People’s Republic of China; 2 Centre for Materials Science, School of Chemistry and Physics, Queensland University of Technology (QUT), 2 George Street, Brisbane, QLD 4001 Australia; 3 Key Laboratory of Advanced Energy Materials Chemistry (Ministry of Education), College of Chemistry, Nankai University, Tianjin, 300071 People’s Republic of China

**Correction to: Nano-Micro Lett (2021) 13:113** 10.1007/s40820-021-00623-5

Due to the technical fault, a wrong version of the paper was uploaded. The content of the article was not affected, but the layout of the article was affected. The original article has been corrected.

