# Supplementary material for: Strongly Coupled 2D Transition Metal Chalcogenide-MXene-Carbonaceous Nanoribbon Heterostructures with Ultrafast Ion Transport for Boosting Sodium/Potassium Ions Storage
Source: Nanomicro Lett. 2021 Apr 22;13:113. doi: 10.1007/s40820-021-00623-5 (PMC8062676; doi:10.1007/s40820-021-00623-5)
Supplement: Supplementary file 1 — Supplementary file1 (PDF 1630 KB) [file 40820_2021_623_MOESM1_ESM.pdf]

Supporting Information for

Strongly Coupled 2D Transition Metal Chalcogenide-MXene-Carbonaceous Nanoribbon Heterostructures with Ultrafast Ion Transport for Boosting Sodium/Potassium Ions Storage

Junming Cao[1,2], Junzhi Li[3], Dongdong Li[1], Zeyu Yuan[1], Yuming Zhang[1], Valerii Shulga[1], Ziqi Sun[2,*], Wei Han[1,*]

[1]Sino-Russian International Joint Laboratory for Clean Energy and Energy Conversion Technology, College of Physics, International Center of Future Science, Jilin university, Changchun 130012, P. R. China

[2]School of Chemistry and Physics, Centre for Materials Science, Queensland University of Technology (QUT), 2 George Street, Brisbane, QLD 4001, Australia

[3]Key Laboratory of Advanced Energy Materials Chemistry (Ministry of Education), College of Chemistry, Nankai University, Tianjin 300071, P. R. China

*Corresponding authors. E-mail: ziqi.sun@qut.edu.au (Z. Sun); whan@jlu.edu.cn (W. Han)

S1 Computational Method and Models

All calculations were carried out by using the projector augmented wave method in the framework of the density functional theory (DFT), as implemented in the Vienna *ab*-initio Simulation Package (VASP). The generalized gradient approximation (GGA) and Perdew–Burke–Ernzerhof (PBE) exchange functional was used. The plane-wave energy cutoff was set to 500 eV, and the Monkhorst–Pack method was employed for the Brillouin zone sampling. The convergence criteria of energy and force calculations were set to 10^{-1} eV/atom and 0.01 eV Å^{-1}, respectively. The $Cu_{1.75}Se$@$Ti_3C_2O_2$@N-doped carbon structure was constructed by packing 3×3 $Cu_{1.75}Se$ (111) slab, 4×4 $Ti_3C_2O_2$ monolayer and N-doped graphene models. The $CoSe_2$@$Ti_3C_2O_2$@N-doped carbon structure was built by packing 2×3 $CoSe_2$(100) slab, $3\times3\sqrt{3}$ $Ti_3C_2O_2$ monolayer and N-doped graphene models. The $NiSe_2$@$Ti_3C_2O_2$@N-doped carbon structure was built by packing 2×2 $NiSe_2$(111) slab, 5×5 $Ti_3C_2O_2$ monolayer and N-doped graphene models. Here, the N-doped graphene model contains three different kinds of N, including pyridinic N, pyrrolic N and graphitic N. A vacuum region of 15 Å is applied to avoid interactions between the neighboring configurations. To explore the interactions between Na(K) and MSe@$Ti_3C_2O_2$@N-doped carbon, including $Cu_{1.75}Se$@$Ti_3C_2O_2$@N-doped carbon, $CoSe_2$@$Ti_3C_2O_2$@N-doped carbon and $NiSe_2$@$Ti_3C_2O_2$@N-doped carbon, the adsorption energies of Na(K) on composite structure were calculated. Here, the adsorption energies (E_a) were calculated by the energy difference of the system after and before adsorption: E_a= [E(Na or K-composite)–n×E(Na or K)– E(composite)]/n, where E(Na or K-composite), E(Na or K), and E(composite) represent the DFT energies of n Na or K adsorbed in composite structures, the energy of a Na or K atom referred to its bulk phase, and the

energy of the clean composite structures. The energy barriers for Na(K) ion diffusion in the composite structures were calculated by the nudged elastic band (NEB) method.

S2 Supplementary Figures and Tables

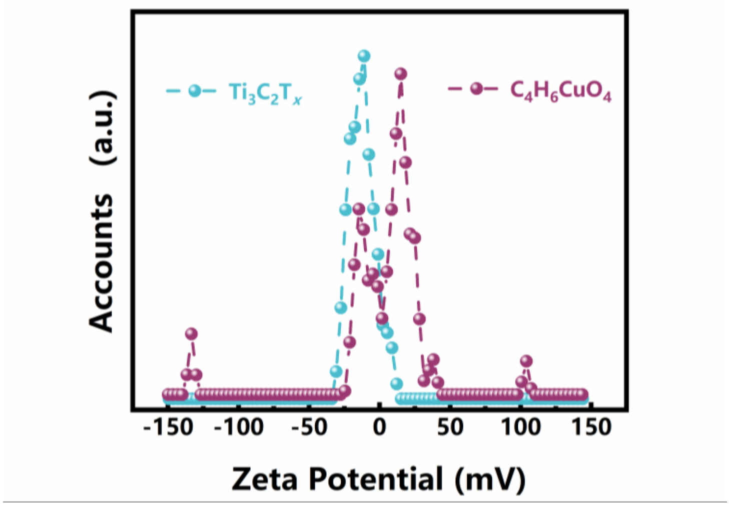

Fig. S1 Zeta potential of $Ti_3C_2T_x$ MXene and $C_4H_6CuO_4$

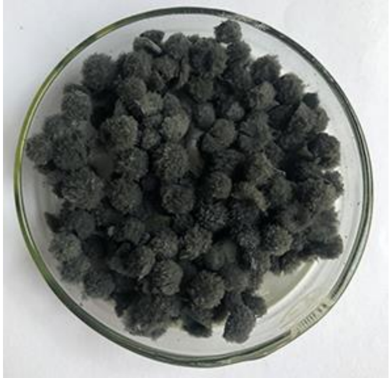

Fig. S2 Digital photograph of $Cu_{1.75}Se$-MXene-CNRib aerogel

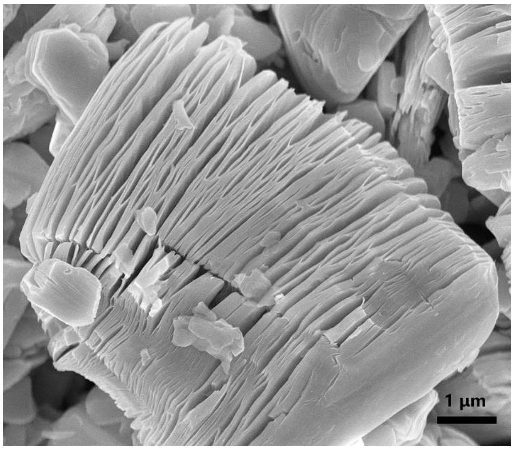

Fig. S3 SEM image of etched multilayered $Ti_3C_2T_x$ MXenes

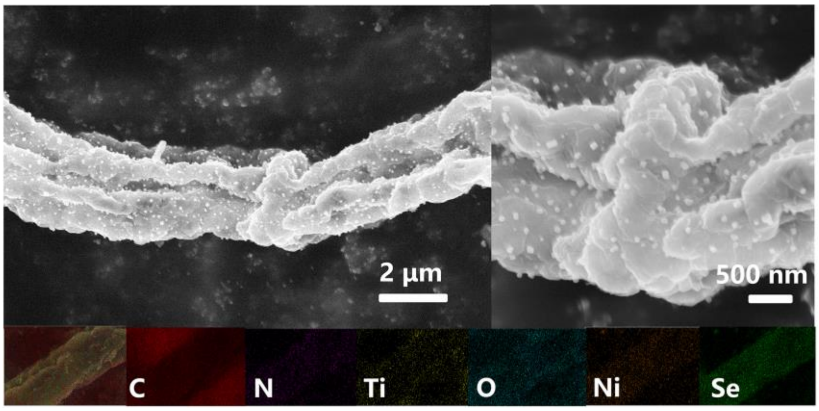

Fig. S4 SEM and magnified images of $NiSe_2$-MXene-CNRib and STEM elemental mapping

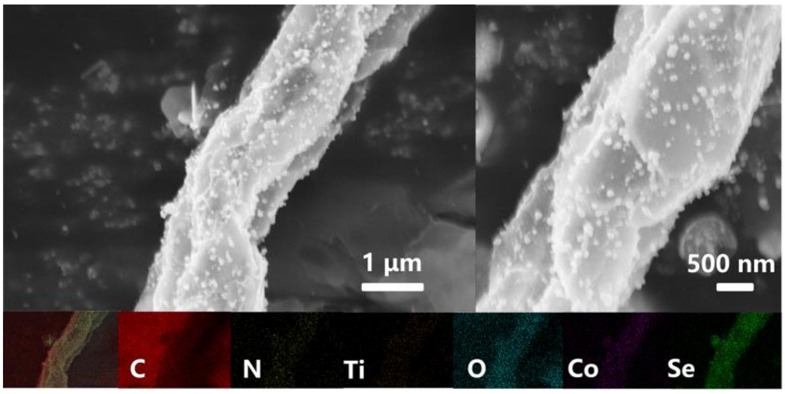

Fig. S5 SEM and magnified images of $CoSe_2$-MXene-CNRib and STEM elemental mapping

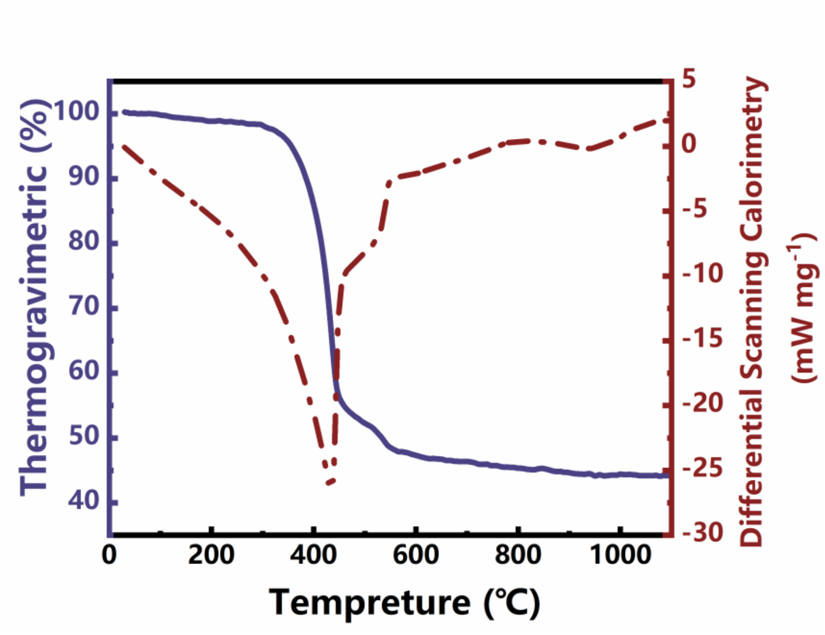

Fig. S6 TG-DSC curve of $NiSe_2$-MXene-CNRib aerogel

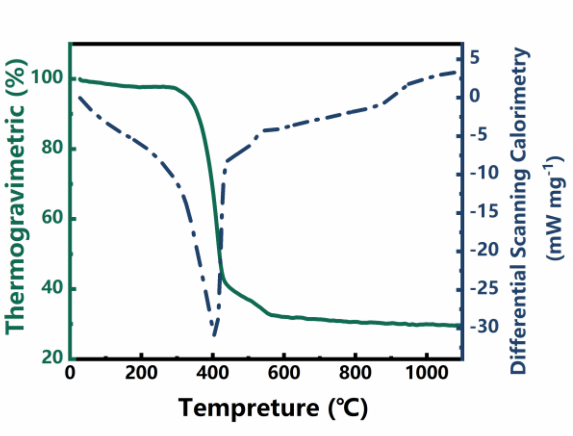

Fig. S7 TG-DSC curve of $CoSe_2$-MXene-CNRib aerogel

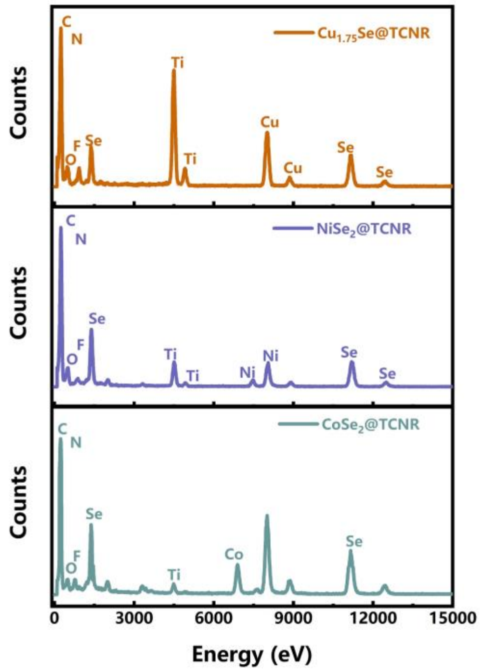

Fig. S8 EDX spectra for $Cu_{1.75}Se$-MXene-CNRib, $NiSe_2$-MXene-CNRib and $CoSe_2$-MXene-CNRib

Table S1 Contents of MSe-MXene-CNRib aerogels

$Cu_{1.75}Se$-MXene-CNRib			$NiSe_2$-MXene-CNRib			$CoSe_2$-MXene-CNRib		
Element	Atomic %	Weight %	Element	Atomic %	Weight %	Element	Atomic %	Weight %
C	64.20	31.97	C	80.43	52.45	C	74.26	58.26
N	4.84	2.81	N	3.28	2.71	N	3.97	2.97
O	5.45	3.61	O	5.62	5.30	O	6.84	4.12
F	0.24	0.19	F	0.27	0.31	F	0.15	0.28
Ti	12.89	25.61	Ti	2.63	7.45	Ti	7.13	7.54
Cu	7.31	19.26	Ni	2.82	8.86	Co	2.42	8.12
Se	5.04	16.51	Se	4.91	22.89	Se	5.23	18.71

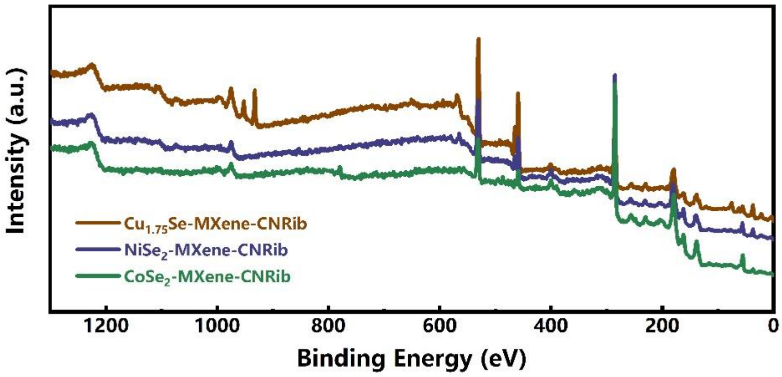

Fig. S9 XPS survey of MS-MXene-CNRib aerogels

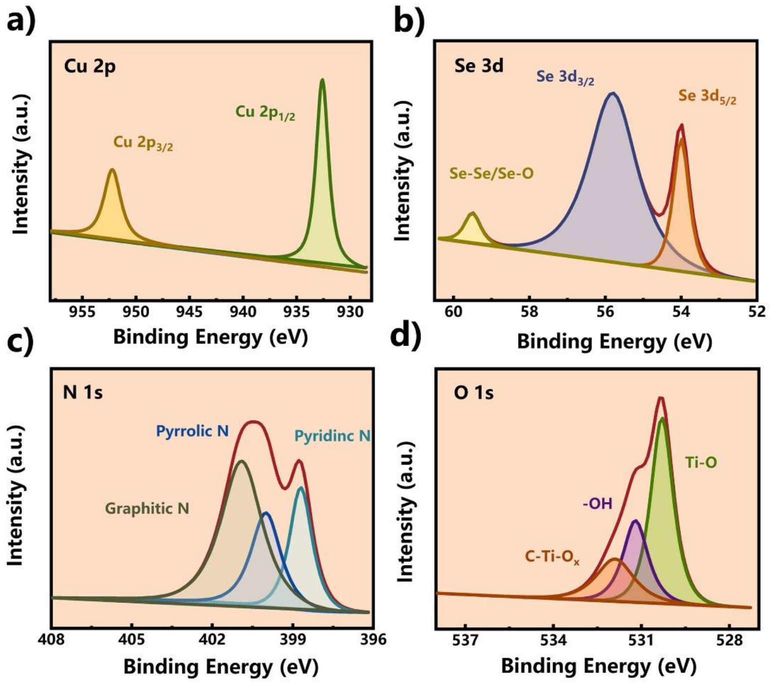

Fig. S10 XPS spectra of $Cu_{1.75}Se$-MXene-CNRib: Cu 2p, Se 3d, N 1s and O 1s

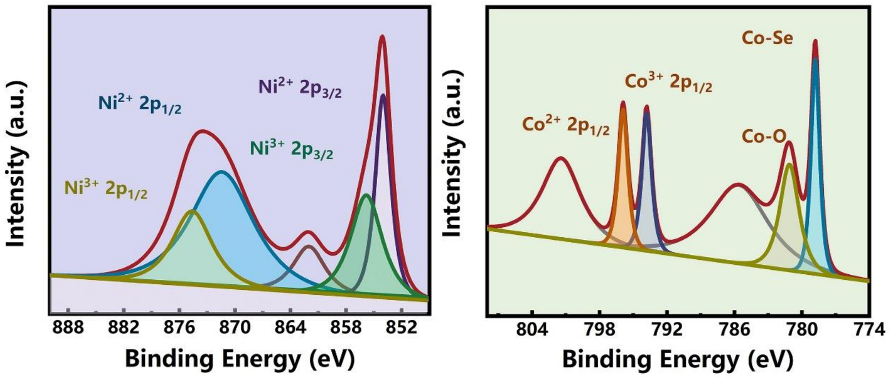

Fig. S11 High-resolution Ni 2p XPS spectra of $NiSe_2$-MXene-CNRib and Co 2p XPS spectra of $CoSe_2$-MXene-CNRib

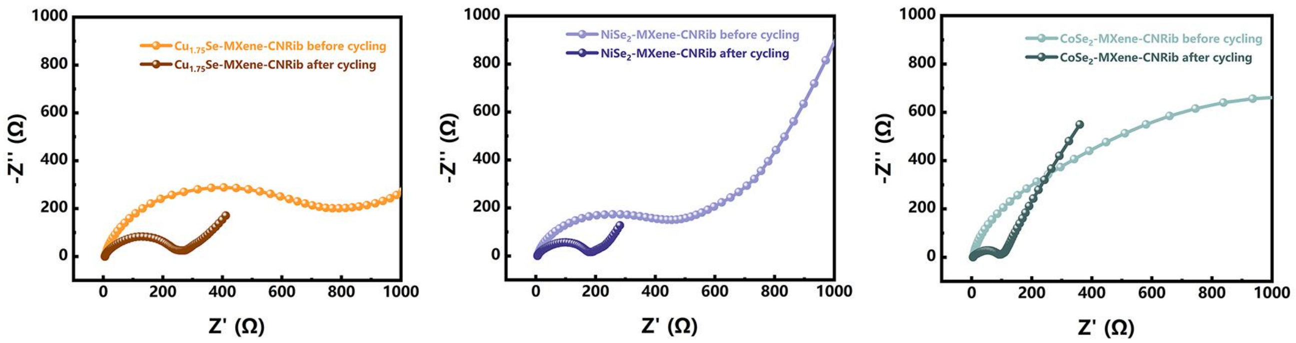

Fig. S12 Electrochemical impedance spectroscopy (EIS) before and after cycling test of $Cu_{1.75}Se$-MXene-CNRib, $NiSe_2$-MXene-CNRib and $CoSe_2$-MXene-CNRib in SIB applications

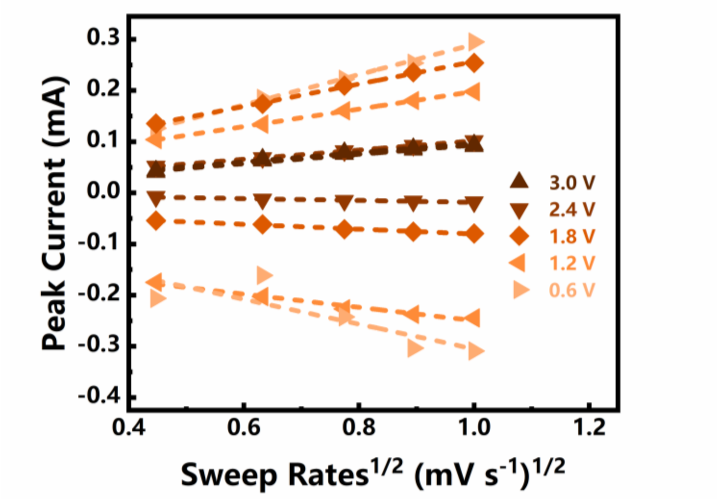

Fig. S13 Relationship between peak current and scan rates$^{1/2}$ at some specific potentials for $Cu_{1.75}Se$-MXene-CNRib

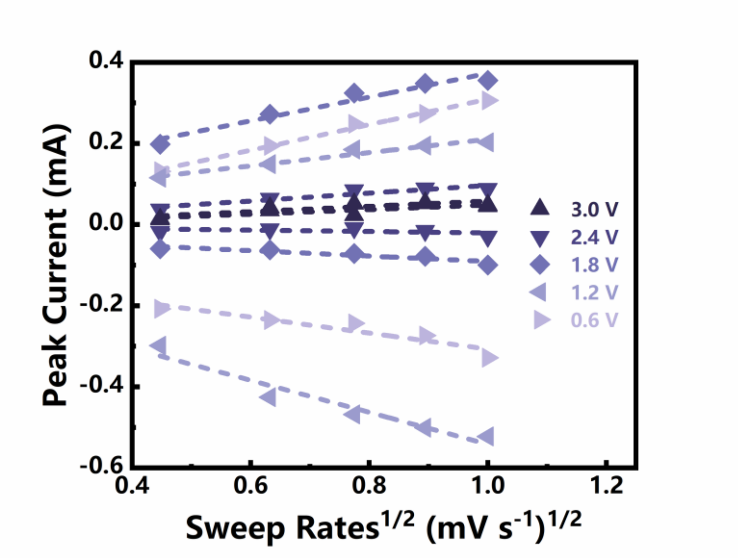

Fig. S14 Relationship between peak current and scan rates$^{1/2}$ at some specific potentials for $NiSe_2$-MXene-CNRib

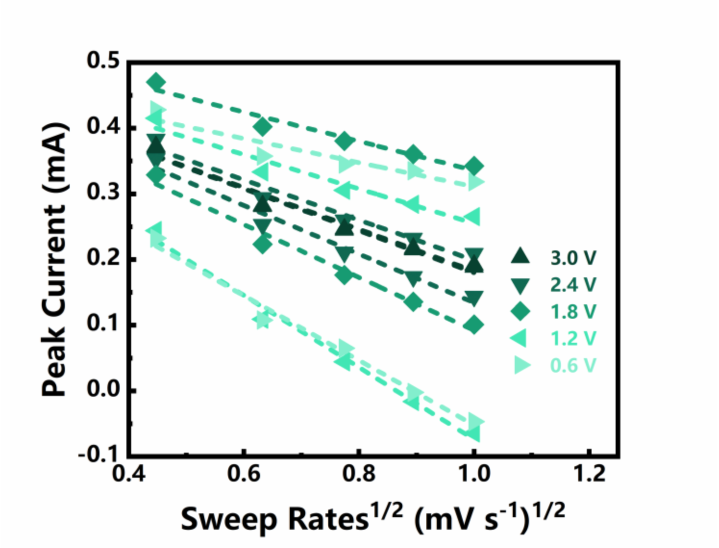

Fig. S15 Relationship between peak current and scan rates$^{1/2}$ at some specific potentials for $CoSe_2$-MXene-CNRib

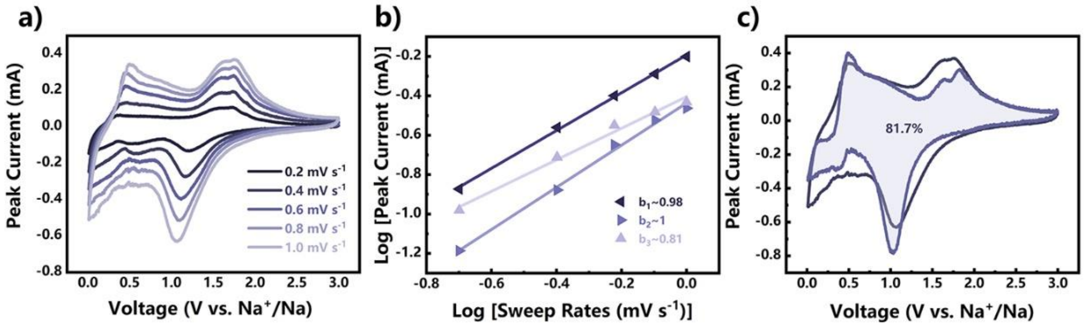

Fig. S16 CV curves at different sweep rates, linear logarithmic relationships between peak current *vs.* various sweep rates and CV curves at 1.0 mV s^{-1} with the shaded area refers to the pseudocapacitive-dominated proportion for $NiSe_2$-MXene-CNRib

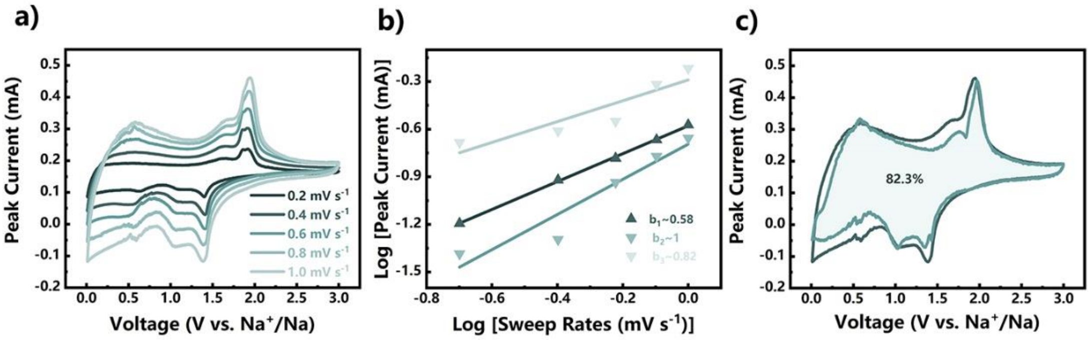

Fig. S17 CV curves at different sweep rates, linear logarithmic relationships between peak current *vs.* various sweep rates and CV curves at 1.0 mV s^{-1} with the shaded area refers to the pseudocapacitive-dominated proportion for $CoSe_2$-MXene-CNRib

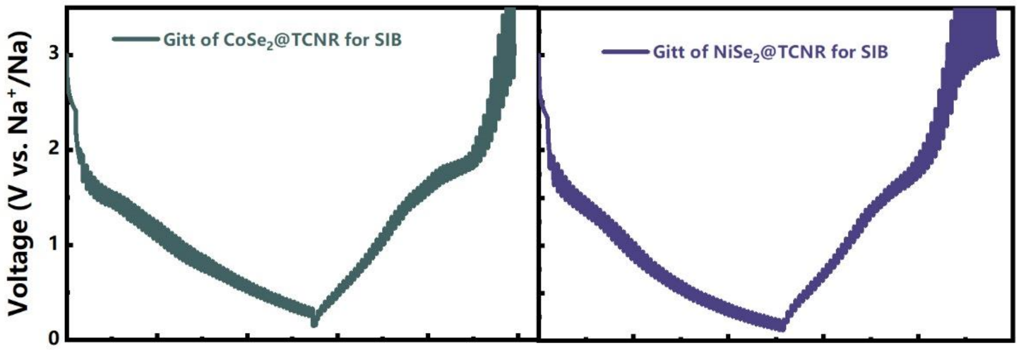

Fig. S18 GITT potential profiles and corresponding Na ions diffusivities *vs.* states of sodiation/desodiation for $CoSe_2$-MXene-CNRib and $NiSe_2$-MXene-CNRib

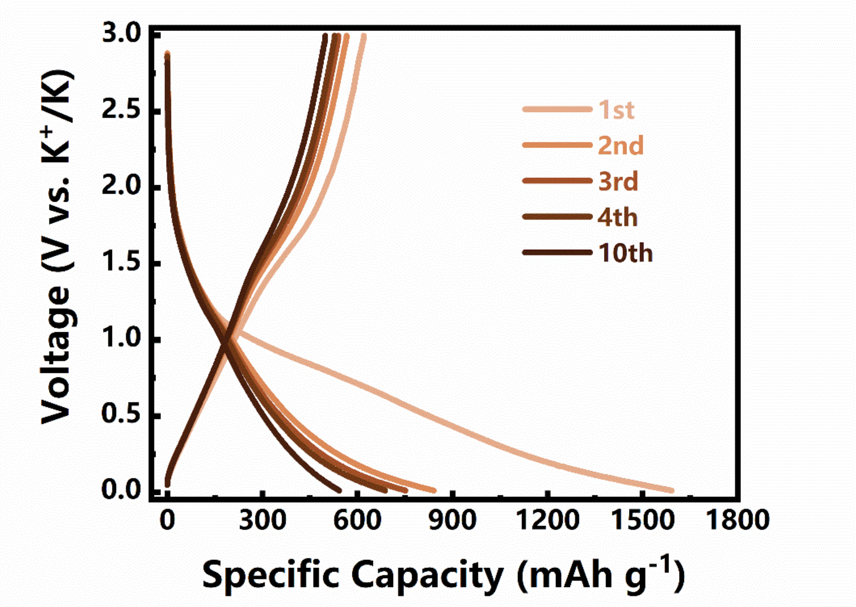

Fig. S19 GCD branches of $Cu_{1.75}Se$-MXene-CNRib hybrids in PIBs

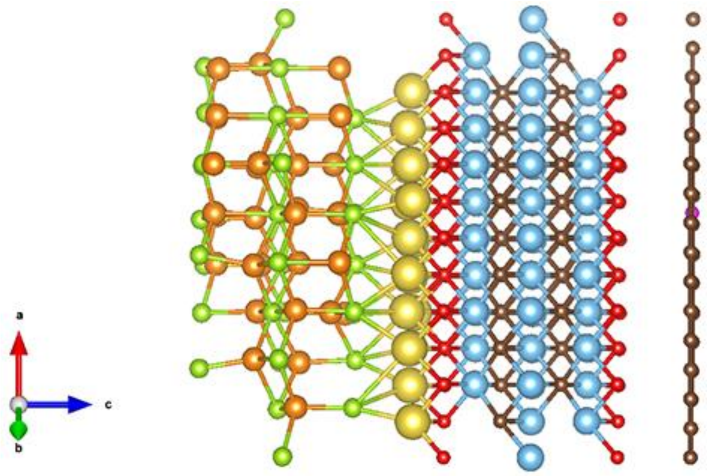

Fig. S20 Side view of Na diffusion sites in $Cu_{1.75}Se$-MXene-CNRib

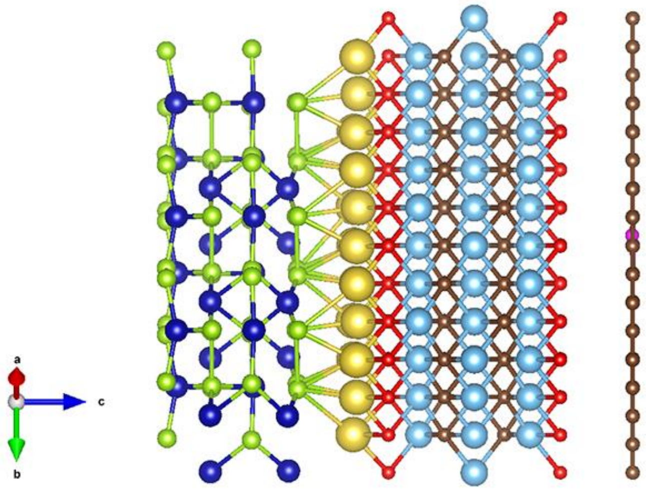

Fig. S21 Side view of Na diffusion sites in $NiSe_2$-MXene-CNRib

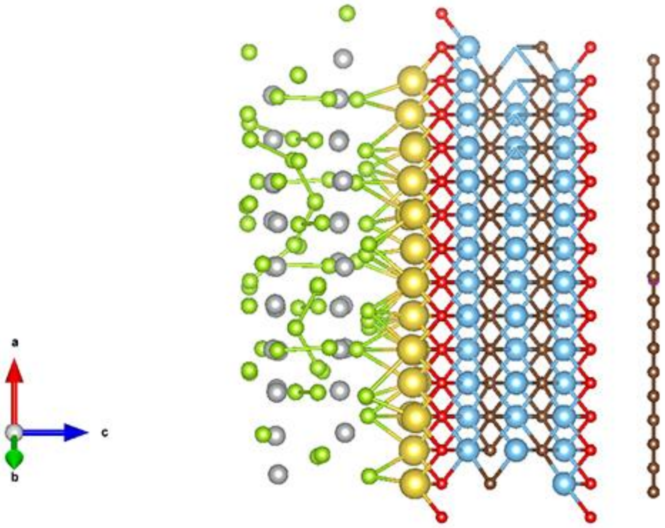

Fig. S22 Side view of Na diffusion sites in $CoSe_2$-MXene-CNRib

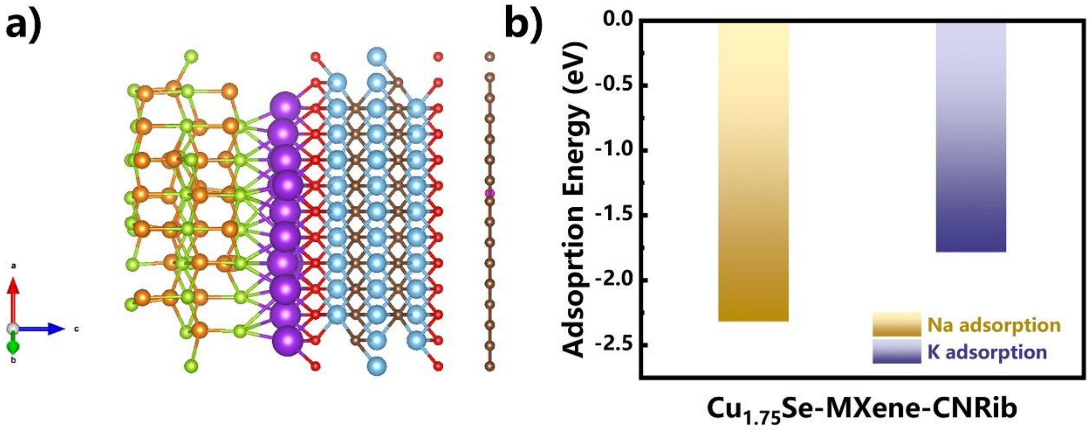

Fig. S23 Side view of K diffusion sites in $Cu_{1.75}Se$-MXene-CNRib and corresponding adsorption energies

Table S2 Summary of main electrochemical properties of $Cu_{1.75}Se$-MXene-CNRib and other anodes for SIBs and PIBs

Materials	Specific Capacity		*b*-values	D_{Na}/D_{K}	Capacitive Contributions at 1.0 mV s^{-1} (%)	Applications	Ref.
	0.1 A g^{-1}	1.0 A g^{-1}					
$Cu_{1.75}Se$-MXene-CNRib	536.3	480.7	~1	10^{-8}	83.5	SIBs	This work
Ti_3C_2/NiCoP	374.8	261.7	N/A	N/A	82 (0.3 mV s^{-1})	SIBs	33
CT-S@ Ti_3C_2	492	358	0.95	10^{-10}	73	SIBs	34
T-MXene@C	257.6	139.5	0.92	10^{-8}	63.2	SIBs	35
CCNA	421.6	389.0	0.98	10^{-11}	77.8	SIBs	14
CNT/$CoSe_2$/C	531	348	0.89	N/A	69	SIBs	17
$Cu_{1.75}Se$-MXene-CNRib	401.3	306.1	0.97	10^{-10}	78.5	PIBs	This work
Sb/Na-$Ti_3C_2T_x$	392.2	~200	0.65	10^{-17}	55.2	PIBs	36
NG/$ReSe_2$/MXene	395.3	215.3	0.86	N/A	80.6	PIBs	37
MXene/MoS_2	233.1	168.2	0.90	N/A	55.8	PIBs	38
PN-PCM	396	218	0.7	10^{-9}	63.9	PIBs	39
$MoSe_2$/MXene@C	355	317	0.93	N/A	89.2	PIBs	24
